# Biological fractionation of lithium isotopes by cellular Na^+^/H^+^ exchangers unravels fundamental transport mechanisms

**DOI:** 10.1016/j.isci.2023.106887

**Published:** 2023-05-15

**Authors:** Mallorie Poet, Nathalie Vigier, Yann Bouret, Gisèle Jarretou, Romain Gautier, Saïd Bendahhou, Vincent Balter, Maryline Montanes, Fanny Thibon, Laurent Counillon

**Affiliations:** 1 Université Côte d’Azur, CNRS, Laboratoire de Physiomédecine Moléculaire (LP2M), Laboratories of Excellence Ion Channel Science and Therapeutics, Nice, France; 2 Oceanography Laboratory of Villefranche (LOV, IMEV), CNRS, Sorbonne University, Villefranche-sur-Mer, France; 3 Université Côte d’Azur, CNRS, Institut de Physique de Nice (INPHYNI), Nice, France; 4 Université Côte d’Azur, CNRS, Institut de Pharmacologie Moléculaire et Cellulaire (IPMC), Valbonne, France; 5 École Normale Supérieure de Lyon, CNRS, Laboratoire de Géologie de Lyon, Lyon, France

**Keywords:** Isotope chemistry, Biological sciences, Biochemistry

## Abstract

Lithium (Li) has a wide range of uses in science, medicine, and industry, but its isotopy is underexplored, except in nuclear science and in geoscience. ^6^Li and ^7^Li isotopic ratio exhibits the second largest variation on earth’s surface and constitutes a widely used tool for reconstructing past oceans and climates. As large variations have been measured in mammalian organs, plants or marine species, and as ^6^Li elicits stronger effects than natural Li (∼95% ^7^Li), a central issue is the identification and quantification of biological influence of Li isotopes distribution. We show that membrane ion channels and Na^+^-Li^+^/H^+^ exchangers (NHEs) fractionate Li isotopes. This systematic ^6^Li enrichment is driven by membrane potential for channels, and by intracellular pH for NHEs, where it displays cooperativity, a hallmark of dimeric transport. Evidencing that transport proteins discriminate between isotopes differing by one neutron opens new avenues for transport mechanisms, Li physiology, and paleoenvironments.

## Highlights

• Biological Fractionation of Lithium Isotopes is debated in the scientific community
• This works shows that membrane transporters and channels transport ^6^Li faster than ^7^Li
• Membrane proteins can separate isotopes that differ by only one neutron
• This has implications in biophysics, physiology, ecology, and paleoclimate reconstruction

## Graphical abstract

**Figure:**
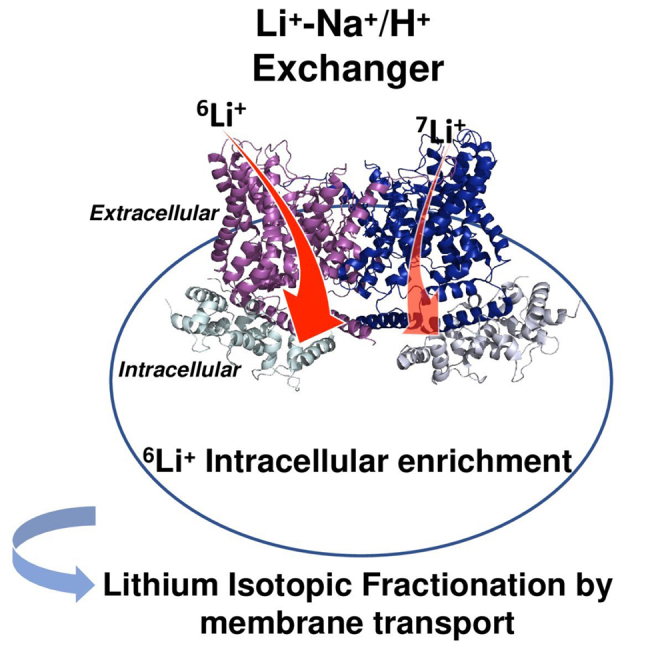

## Introduction

**Figure 1:**
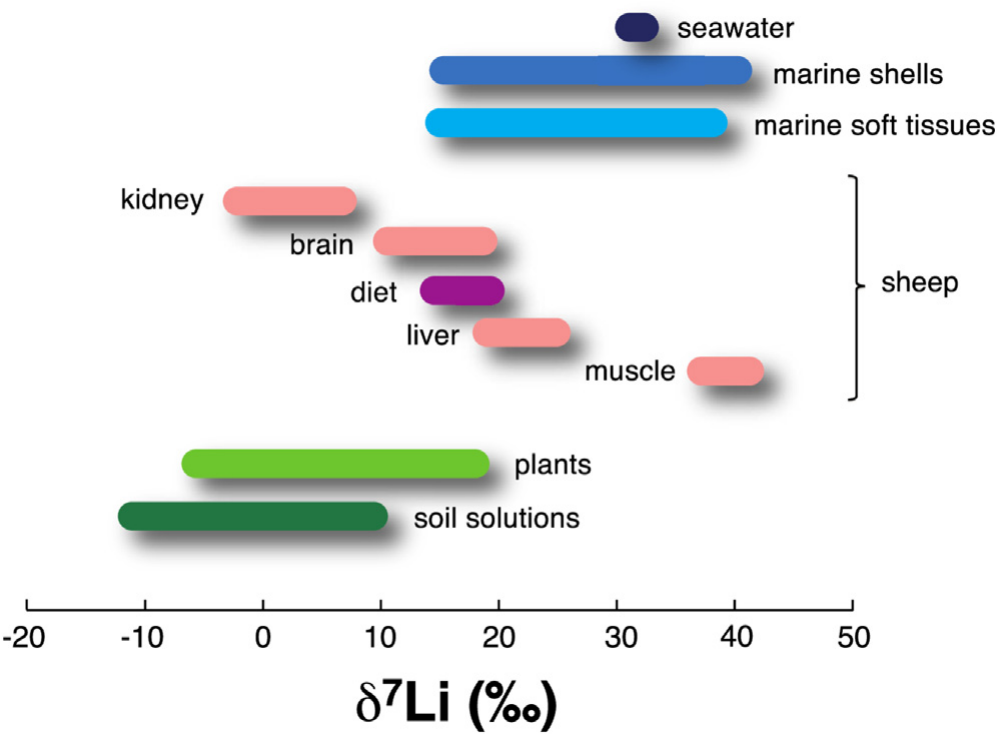
Compilation of published Li isotope compositions measured in biologic materials and their environments By convention, Li isotope compositions are expressed in δ^7^Li (‰) = (^7^Li/^6^Li)/(^7^Li/^6^Li)_LSVEC_ – 1) x 1000, LSVEC being the international standard). Biologic samples are: in green, terrestrial plants and corresponding soil solutions^12^^,^^13^ in pink, organs of model mammals (sheep), and corresponding diet in purple^1^; in blue, living and modern shells produced by marine organisms, and soft tissues of marine species,^6^^,^^14^^,^^15^ and seawater in dark blue. Note that the ocean is currently homogeneous in terms of Li concentration (26 μM) and δ^7^Li (31.2‰ +/− 0.3‰).^3^ The range displayed by all the biologic samples is similar as the one estimated for the global Earth.^3^

Evidence for large variations of Li isotopes (reported as δ^7^Li (‰)= ([(^7^Li/^6^Li)/(^7^Li/^6^Li)_lsvec_] – 1) x10^3^), with lsvec as the international standard) in biological samples is compiled in Figure 1A, as illustrated by the difference between the various organs of a mammal model and its diet.^1^ Li isotopes vary also significantly in modern and fossil carbonate shells, relative to homogeneous seawater,^2^^,^^3^^,^^4^^,^^5^ and recent studies^6^^,^^7^^,^^8^ exhibit similar isotopic variations in soft and calcified tissues of marine organisms. Figure 1 highlights that the range displayed by biologic materials is in fact similar as that estimated for the global Earth (∼50‰). Another line of evidence of the biological control on Li isotopes comes from studies focusing on the relative distributions and biological effects of pure ^6^Li and ^7^Li related to a therapeutic use,^9^^,^^10^^,^^11^ suggesting a higher diffusivity of ^6^Li than ^7^Li. Also, the results contained in Figure 1 indicate that Li isotopes accumulate differently in organs and tissues where they can exert distinctive biological effects.

The main way for an alkali cation, such as Li^+^ to enter and accumulate in cells is to be transported across their membranes by ion transporters and/or by channels.^16^^,^^17^ These proteins are embedded in the membranes of all cells and allow the transport of ions that could not otherwise cross the lipidic bilayer barrier. Ion channels allow the passive flow of ions, following their electrochemical gradients, while transporters use energy-active conformational changes to accumulate ions or other solutes, often in opposition to their concentration gradients. Because cells have a negative membrane potential at rest, Li can enter through various ion channels, but can also be actively translocated through ion transporters. In particular, as it is situated between H and Na in the first column of the periodic table, Li is efficiently transported inside cells by Na^+^/H^+^ exchangers (NHEs) of the SLC9 gene family, with rates and affinities comparable to Na, their physiological extracellular cation.^18^ For example, NHE1 Km for Lithium is 9 mM to be compared to 16 mM for Na^+^, indicating that NHE1 does not display any significant selectivity between these ions. NHEs are therefore excellent candidates for providing a molecular mechanism for the biogenic fractionation of Li isotopes.

## Results

### Ubiquitous, apical, and vesicular NHEs fractionate Li isotopes

**Figure 2:**
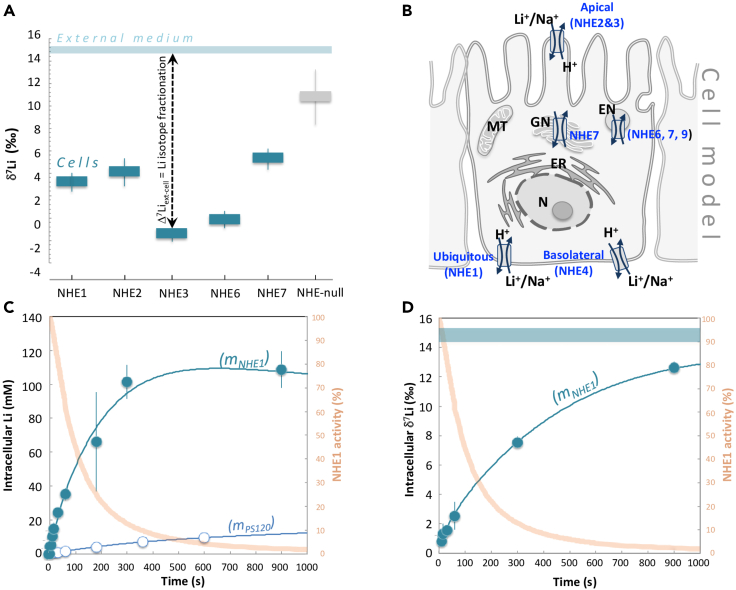
Measurements of Li isotopic fractionation by Na^+^/H^+^ exchangers and channels (A) Li isotope fractionation by Na^+^/H^+^ exchangers. In blue, δ^7^Li values measured in fibroblast cells expressing NHE1, NHE2, NHE3, NHE6, and NHE7, after 1 min of Li uptake. Li uptake experiments were also performed using NHE deficient PS120 fibroblasts^19^ (in gray, NHE-Null). The light blue bar displays the constant δ^7^Li value measured for the external Li uptake solution (15 mM Li). All experiments were performed at 37°C (See Methods in supplemental information). Note the large Li isotopic fractionations (Δ^7^Li) between NHE-expressing cells and the external medium solution. Error bars represent ± SEM. (B) Schematic representation of the cellular localization of NHEs. Li^+^ is an alkali element that is essentially mobile in the outer and inner cellular media. NHE1 is a ubiquitous transporter involved in pH and volume regulation. It is expressed mostly basolaterally in epithelia. NHE2 and NHE3 are apically expressed in epithelial cells, while NHE6 and NHE7 are mostly intracellular and expressed in the Golgi Network and endosomal compartments. N: Nucleus, ER: Endoplasmic Reticulum, GN: Golgi Network, MT: Mitochondria. (C) NHE1 kinetics of Li transport. In dark blue, Li concentrations measured in NHE1 expressing fibroblast, as a function of the external Li uptake duration. The extracellular solution is the same as in Figure 1A (15 mM Li). In light blue, are reported the same experiments for PS120 cells (NHE-Null). Blue lines (m_NHE1_, m_PS120_) display the transport model results fitting all data points (see text and supplemental information). In orange is shown the measured NHE1 activity as a function of time (see supplemental information Methods). Error bars represent ± SEM. (D) NHE1 kinetics of Li isotopes transport. δ^7^Li values (in blue) measured in NHE1 expressing fibroblast as a function of the external Li uptake duration. The blue line (m_NHE1_) displays the NHE1 transport model results, fitting well all data points (see text and supplemental information). The external solution (medium) is the same as in Figure 1A, with a constant δ^7^Li value over the experiment duration. In orange is shown the measured NHE1 activity as a function of time (see Methods). All experiments are performed at 37°C. At 60 s, experiments were reproduced at 20°C (supplemental information). Error bars represent ± SEM.

We first performed various Li uptake experiments in NHE-deficient fibroblasts,^19^ in which we stably and individually expressed different human NHEs using classical gene transfer techniques and selection^20^^,^^21^^,^^22^ (supplemental information). The intracellular δ^7^Li value is measured after 1 min of Li uptake in cells expressing, respectively, the ubiquitous NHE1, the epithelial NHE2^23^ and NHE3,^24^ the vesicular NHE6^25^ and NHE7^22^ (both expressions directed toward the plasma membrane as in^22^), as well as in the NHE-null (i.e. PS120,^15^) cells (Figures 2A and 2B). Compared to the extracellular medium (δ^7^Li = 15 ± 0.3‰), all cytosolic contents of NHE-expressing cells show strong ^6^Li enrichments (lower δ^7^Li values), with the highest and the lowest one for NHE3 (by −15.4‰) and NHE7 (by −10‰), respectively (Figure 2A). The NHE-null PS120 cell line exhibits a minor but non-negligible isotopic fractionation, with an intracellular δ^7^Li value of −4.4‰ lower than the external solution. Thus, NHE-expressing cells, whose exchangers internalize Li in response to cytosolic acidification, display much lower δ^7^Li values than those of equivalent cells, which are not expressing any of those transporters. We, therefore conclude that Li isotopes are strongly fractionated by ionic transport through cell membrane. Given that the only difference between these cell lines is the expression of a specific NHE at the membrane, the large isotopic variations exhibited by the NHE-equipped cells are due to active transport performed by the NHEs.

### Biological Li isotope fractionation is driven by kinetics

We chose NHE1 as a model to quantify biological Li isotopic separation kinetics because it is both well described and present in all eukaryotic cells.^18^ For this, we measured fast kinetics of total Li transport in parallel with its isotopic fractionation (supplemental information). In NHE-null cells (PS120), Li enters passively through ion channels, and this can be evidenced and measured using electrophysiological techniques (Figure S1). Accordingly, Li accumulation followed a two-time scale kinetic (Figure 2C, Data S2), accurately fitted with a Goldman-Hodgkin-Katz equation^16^ (describing the reversal potential across a cell membrane), modified to take into account the variations in membrane potential due to the entry of the positive charges provided by Li^+^ itself (supplemental information). This was accompanied, at 1 min, with a relatively small isotopic fractionation of −4.4‰ relative to the external medium (Figure 2A).

In NHE1-expressing cells, rates of Li^+^ accumulation show a short exponential corresponding to pre-steady-state, followed by a quasi-linear uptake corresponding to steady-state behavior. Finally, Li intracellular concentration stabilizes over long durations (Figure 2C). Between 5 and 30 s intracellular δ^7^Li remained low, at 1.2 ± 0.3‰ on average, compared to 15 ± 0.3‰ for the extracellular medium (Figure 2D, Data S1). This significant isotopic fractionation (of −13.8‰), in favor of the lightest ^6^Li isotope, occurred at maximal NHE1 activity and demonstrates the potential of the ubiquitous NHE1 to strongly fractionate Li isotopes, while it maintains intracellular pH. The magnitude and evolution of the cell δ^7^Li value as a function of time rule out a significant contribution of differential sequestration of the two Li isotopes by a hypothetic cytoplasmic component (such as metallothionein in the case of copper isotopes for instance^26^^,^^27^ and are consistent with the fact that monovalent cations, such as Li^+^, Na^+^, and K^+^ are extremely mobile within cells.^16^

### pH induced and dose-response of Li isotope fractionations reveal cooperativity

As NHEs interact with both the external and cytosolic medium to transport H^+^ and ions, the corresponding transport rates depend on the external solution concentrations of these ions, as well as on the intracellular pH. Therefore, measuring those rates at different ionic concentrations and pH (i.e. dose-responses) provides valuable information, which are complementary to those provided by kinetics.

**Figure 3:**
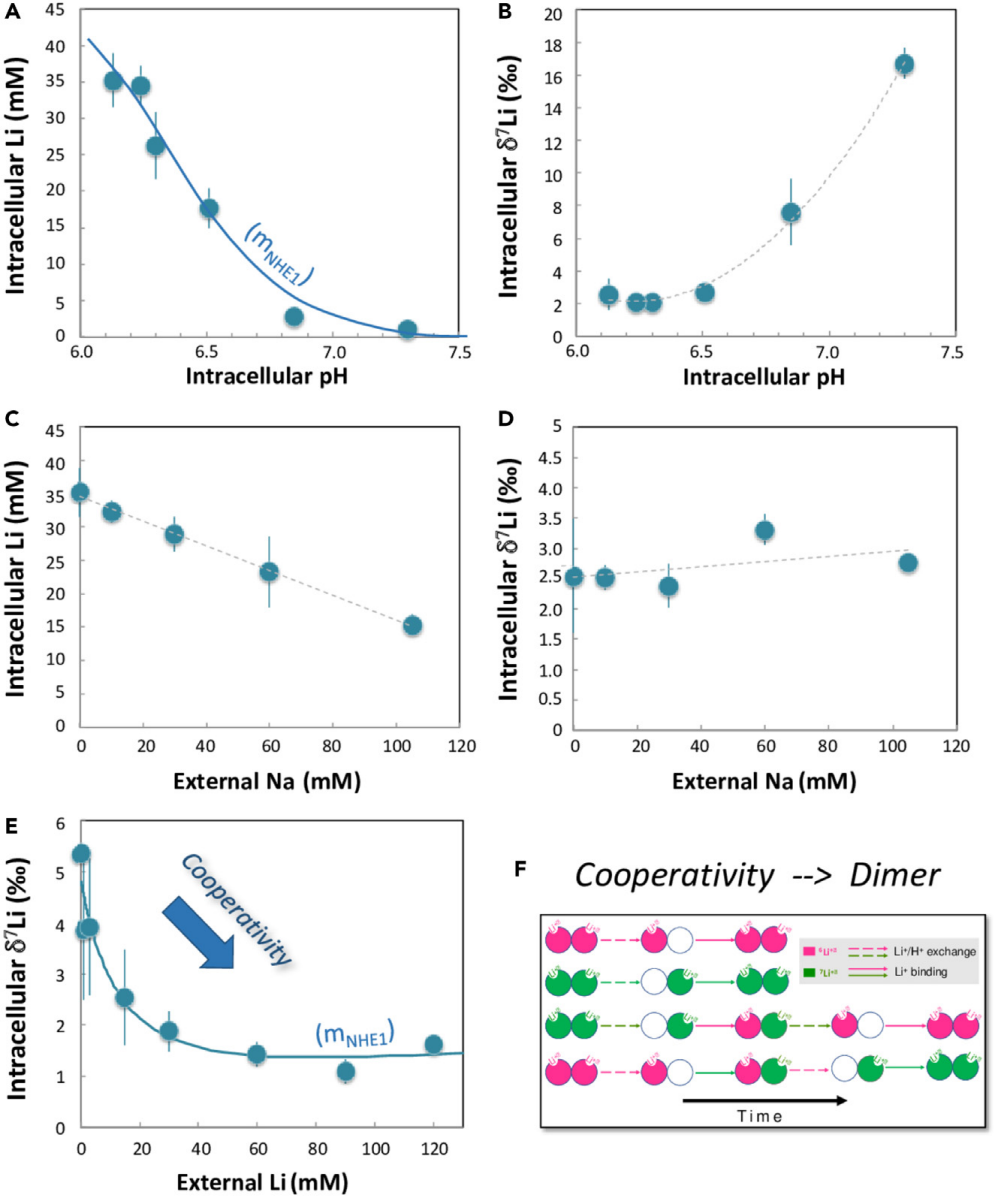
Dose responses for Li isotopic fractionation by NHE1 (A and B) Intracellular pH dependency on Li isotopic transport by NHE1. Cell Li concentration (a) and δ^7^Li value (b) as a function of intracellular pH. Li uptake was performed during 1 min, at various cytosolic pH for NHE1-expressing fibroblasts. See Methods in supplemental information for details on cell pH control and calibration. The Li transport and associated Li isotope fractionations are maximum at the lowest intracellular pH when NHE1 is at its maximal rate. Total Li uptake was fitted using the MWC equation for a dimeric NHE1,^28^ with K_h_ = 0.17 10^−7^ M, K_l_ = 36 10^−7^ M, L_0_ = 878.6 ± 35.3 (4.02%), Vmax = 55.4 ± 0.83 (1.51%) and R^2^ = 0.981. Error bars represent ± SEM. (C and D) Dose response for extracellular lithium and cooperative behavior. δ^7^Li values of NHE1-expressing cells were measured after 1-min incubation at different extracellular Li concentrations (supplemental information). Note the steady decrease of δ^7^Li as extracellular Li increases, highlighting the unexpected cooperativity of the Li transport process d. Simplified scheme illustrating how a dimeric NHE1, binding external two ^6^Li^+^ (in pink), two ^7^Li+ (in green) or a mixing of both, before exchanging them with H^+^ will favor the lighter isotope transport. Error bars represent ± SEM. (E and F) Impact of external Na concentration on Li isotopic transport by NHE1. Intracellular Li concentration (c) and δ^7^Li value (d) as a function of external Na concentrations. The presence of Na in the external medium decreases the Li transport, due to competition effects. In contrast, the cell δ^7^Li value evolves little, showing a slight increase only. Error bars represent ± SEM.

We subsequently determined the Li isotope fractionation between the external medium and cells (Δ^7^Li) - after 1 min—at different H^+^ intracellular concentrations (different cytosolic pH). As NHE1 kinetic is allosterically regulated by cytosolic pH,^28^ we expected the Δ^7^Li value to be larger in magnitude at more acidic pH, when the transport activity is stronger (supplemental information). In contrast, at higher intracellular pH, Li isotope fractionation was expected to be smaller, because NHE1 is slower, and passive transport by ion channels would take over. This is indeed what we observed (Figures 3A and 3B, Data S5): the largest differences between cells and external solution (Δ^7^Li = -12.5 ± 0.9‰) are measured for the lowest intracellular pH (Figure 3B). As intracellular pH dictates NHE1 rates, these results indicate that Li isotopic fractionation is kinetically driven by intracellular proton.

Experiments performed at various extracellular Li concentrations, from 1 to 120 mM, yielded an unexpected result (Data S3). Total Li uptake displayed a typical Michaelis-Menten saturation, with a fitted Km value of 9.94 ± 0.71 mM (r^2^ = 0.99) in accordance with published data (Data S3). In contrast, the isotopic ratio of transported Li exhibited interesting variations, starting from a δ^7^Li value of 5.4‰ (for an extracellular Li concentration of 0.3 mM), and then steadily decreasing to reach a value around 1.4‰, for media Li contents greater than 60 mM (Figure 3C). This decrease reveals that, as the external Li concentration increases in total, ^6^Li enhances its own transport against ^7^Li. Such a result is the hallmark of positive cooperativity, which occurs here for the transport of an isotope. As NHE1 is a dimer that displays cooperativity for internal H^+^(Lacroix et al., 2004), cooperativity for external cations at steady-state was expectable, but has been so far impossible to detect, with the only previous evidence coming from daring pre-steady-state experiments.^29^ Here, this phenomenon is unraveled by the unprecedented high resolution given by Li isotopic measurements by MC-ICP-MS (supplemental information).

### Mathematical derivation of Li isotopic fractionation by a dimeric exchanger

In order to reach a fine mechanistic understanding of our experimental results, we developed a mathematical framework for isotopic transport, based on the kinetics of Li and Na transport by NHE and ion channels. ^6^Li and ^7^Li differential transport in NHE1-expressing cells can be calculated by summing the rates of their passive entry through channels, with those of NHE active electroneutral transport. The negative membrane potential triggers the electro-osmotic accumulation of Li species. This is then counterbalanced by the main electrogenic ions, namely by an exit of K^+^ and an entry of Cl^−^ proportional to their respective permeabilities, which, in turn, modify the membrane potential according to the net charge balance. The corresponding Goldman-Hodgkin-Katz flux equations derived from^16^ are developed in the supplemental information.

As NHE1 is electroneutral, its transport rate only depends on the inner and the outer transported cations. On each protomer, we formalized NHE1 transport in three simplified steps, in accordance with the Na^+^/H^+^ exchange elevator model for ion translocation^30^^,^^31^: (1) Li^+^ and H^+^ ions bind NHE in fast pre-equilibria (2) a translocation mechanism occurs that exchanges the NHE-bound ions, (3) NHE is recycled to the initial state, concomitantly extruding the proton to the outer medium and Li^+^ to the inner medium. We described each elementary transformation by a microscopic kinetic constant of the appropriate order and minimally simplified the overall scheme, assuming that the transformations occurring within the transmembrane part of the transporter form fast pre-equilibria.

The kinetic isotopic effect on each microscopic constant produced different rates for the two isotopes. We next introduced the dimeric NHE using the “flip flop” mechanism^32^ described by Otsu et al.^29^ in which each protomer alternatively performs one elementary exchange. Thus, a protonated NHE1 sequentially binds a ^6^Li or a ^7^Li on a first protomer, and then a second ^6^Li or ^7^Li on the other protomer before it can start to exchange ions^29^ (Figure 3D). Following this, a dimer having bound two ^6^Li (E_66_) has the shortest and fastest pathway to regenerate E_66_ after having released one ^6^Li (Figures 3D and supplemental information). In contrast any conversion of a dimer having bound two ^7^Li (E_77_) into E_66_ requires a larger number of steps. Without introducing any additional *ad hoc* change (e.g. affinity or conformation), this combinatorial effect leads to a first come-first serve mechanism, in which ^6^Li transport enhances itself, which is what we observe (Figure 3C).

A complete derivation for isotopic transport is given in the supplemental information. As in previous modeling studies of ionic transport,^33^ this approach is mechanistic as it is data driven, and based on physical laws and kinetics of ion transport. It is of note that the resulting set of equations fits (i) the total Li intake (Figure 2C), (ii) the isotope fractionation kinetics (Figure 2D), and (iii) the cooperativity of ^6^Li transport within the dimer (Figures 3C and 3D), which supports the relevance of our approach. Taken together, modeling NHE Li isotopic transport shows both how the H^+^ gradient drives the isotopic separation and that the sequential binding steps of a dimeric NHE1 trigger positive cooperativity when the external Li concentration increases. As expected, it also shows that the isotope fractionation reaches a saturation value at high external Li concentrations (≫100 mM). The model determines, for NHE1, a maximal isotopic fractionation between cell and solution of −21.3‰ (±-2‰). This calculated value is slightly greater than what we found after a few seconds of activity, pointing to the very rapid turnover of NHE1, which changed the cell pH after 5 s only. Taken together, NHE performs a fractionation that is 6 times more efficient than electro-osmotic fractionation by ion channels (2–3‰) at the same temperature (supplemental information).

We also show that NHE1 mediated-isotope fractionation is very moderately decreased when Na^+^ concentration increases (Figure 3E and 3F, Data S4). This is an interesting difference compared to the observed effect of the inhibitor Cariporide,^34^ which limits the total Li NHE1 transport by competition, but cannot affect the measured Li isotopic fractionation occurring when Li is bound and transported (supplemental information). Na^+^ behaves differently as it is able to both bind and be translocated, such as Li^+^. Hence in a solution containing both extracellular Li and Na, the dimeric NHE1 is able to bind two Na^+^ ions (in this case no Li^+^ transport occurs), two Li^+^ ions (in this case NHE1 fractionates as described above), or one Na^+^ and one Li^+^ (and in this case NHE behave as a monomer with respect to Li^+^). Consequently, when Na^+^ concentration increases, the proportion of “mixed” versus “fully bound to Li” NHE increases as well, thereby decreasing the cooperative effect and slightly the intracellular δ^7^Li value (Figure 3F).

### Molecular dynamics simulation of Li transport by NHE1

**Figure 4:**
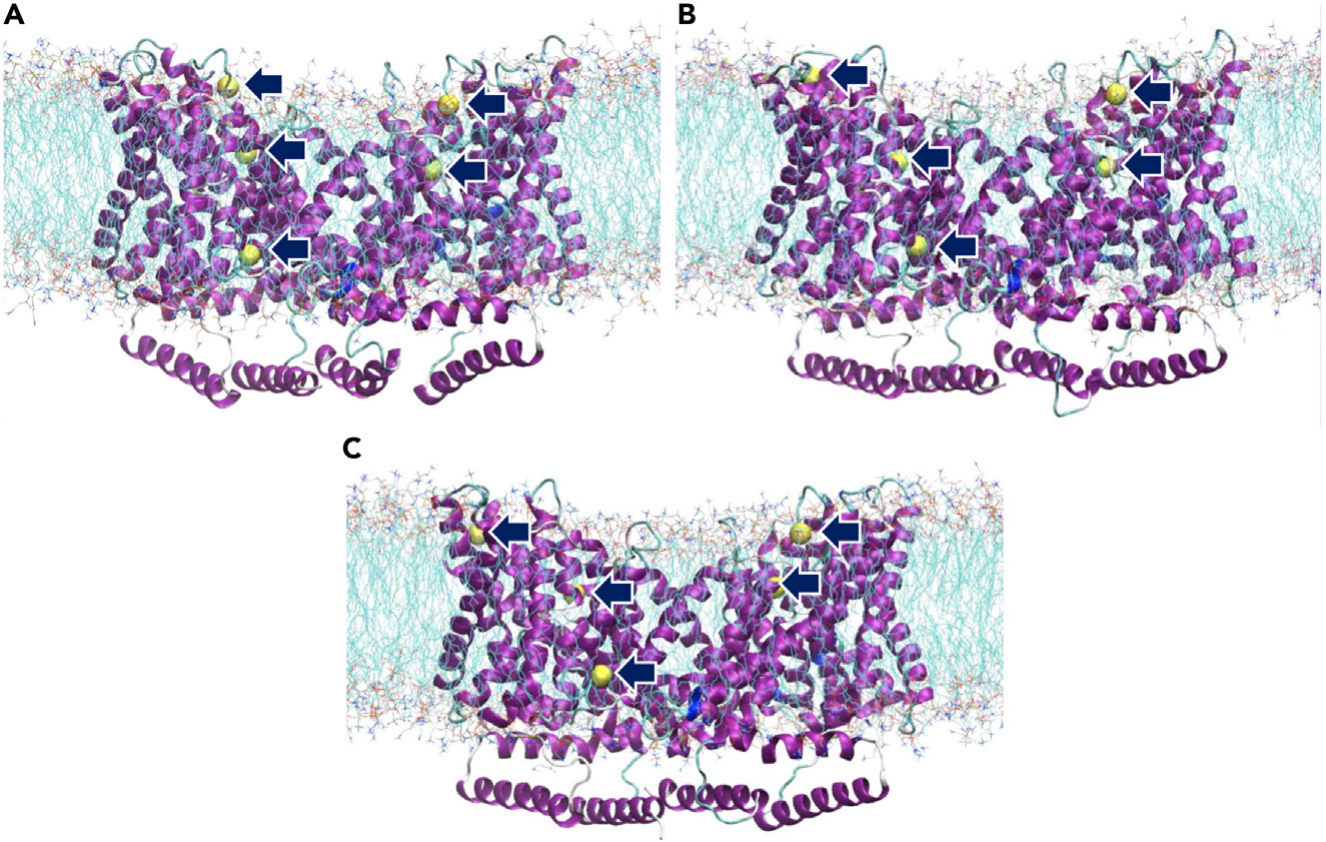
Extracts of the MD simulation movie showing NHE1 symmetrical dimer (in purple), with lipids (POPC, in blue) (A–C)H_2_0 and Li^+^ (in yellow) along the ion translocation pathway (see Video S1). Li^+^ ions at low potential energy sites within the translocation pathway are marked by blue arrows. A, B, and C images can be seen at 3.8, 12.6, and 23.2 s (movie time) in Video S1, respectively Note the very dynamic structure of the proteins and the different positions of the lithium ions.

To visualize the relation between the mechanism of Li transport and its isotopic fractionation, we ran molecular dynamics (MD) simulations (Gromacs MD software,^35^ 250ns at 310K with images every 100ps, see the STAR Methods) of Li^+^ ions within the NHE1 structure, built from its PDB coordinates^31^ within a lipid membrane in a water box containing Cl^−^ and Na^+^ ions that were replaced by Li^+^ close to the protein. As no force fields exist for the isotopes of the same ion, we used the existing CHARMM36 force field to run the MD simulations. Figure 4 and Video S1 show that during the 250ns simulation, the atoms of the protein and surrounding lipids constantly vibrate but do not sample very different conformational states. The lithium ions occupy different low potential energy locations within the transporter, consistent with the sites where most of the mutations affecting binding and transport have been found (for review see^36^). To the best of our knowledge this is the first time that we can visualize those microscopic states of Li occupation that delineate the ion translocation pathway within the NHE1 structure.

Video S1. Molecular dynamics simulation of Li^+^ ions in NHE1 structure, associated with Figure 4 “Extracts of the MD simulation”, related to STAR Methods “Molecular Dynamics (MD) simulations” The movie shows the NHE1 structure (blue/purple ribbons representing NHE1 alpha helices) built from the PDB coordinates of the NHE1 dimer complexed with CHP (PDB 7DSX), within a lipid membrane in a water box containing Cl^−^ and Na^+^ ions. The latter were replaced by Li^+^ (represented by yellow spheres) close to the protein (CHARMM36 force field). The movie shows MD simulations (Gromacs MD software) ran for 250ns with a sampling scale of 100ps. We can observe that lithium occupies three main low potential energy locations within the transporter, thus delineating the ion translocation pathway. At the simulation sampling time, lithium movements have an important vibrational component within their sites and can sometimes translocate from one to another, thereby crossing an energy barrier within the translocation pathway

At the simulation sampling scale (100ps), Li ions display an important vibrational component within each of their coordination sites and can be seen translocating from one to another (Video S1), thereby crossing a local energy barrier within the translocation pathway. As mentioned previously, no force field exists to simulate two different isotopes of the same ion. Nevertheless, this MD simulation makes obvious that if one isotope has a greater vibrational energy it will gather translocation speed each time it will cross a local energy barrier (see discussion below).

## Discussion

**Figure 5:**
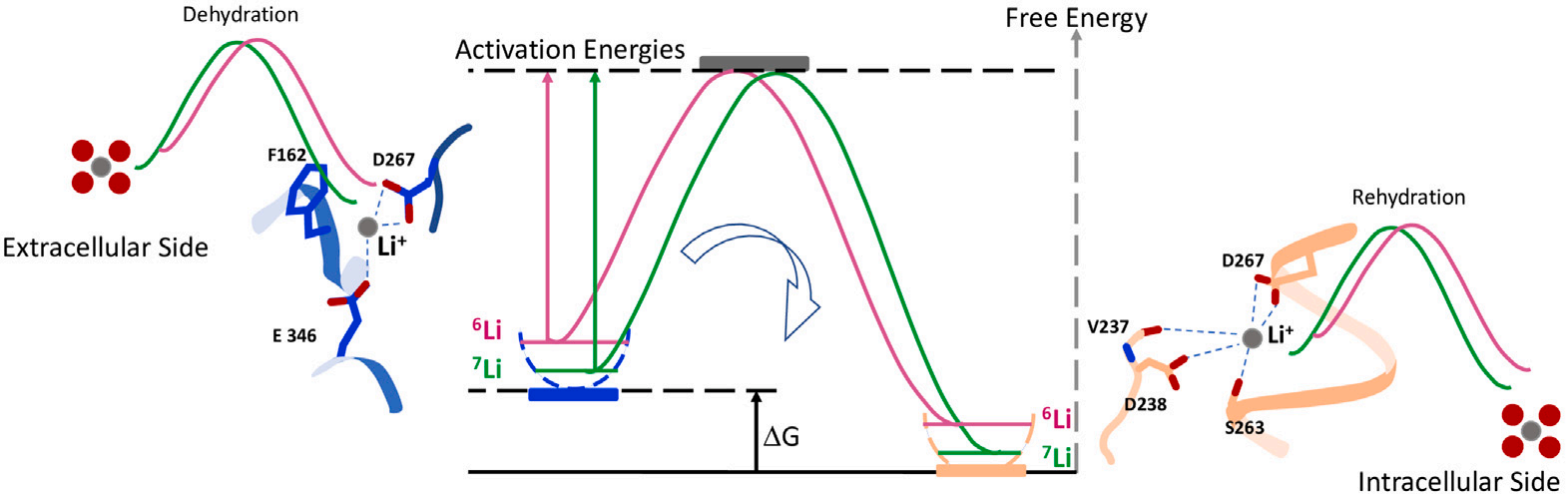
Schematic illustration of the NHE1 isotopic effect During ion dehydration/hydration and translocation between outward and inward-facing local energy grooves, Li^+^ ions have to cross microscopic activation energy barriers that are smaller for the lighter ^6^Li^+^ (pink) than for the slower and heavier ^7^Li^+^ ion (green). The outward facing conformation (blue) is extrapolated from the NHE1 guanidinium binding site, the cytosol facing conformation (light brown) corresponds to the thallium binding, as described both in.^31^

Overall, our study demonstrates that ion transport proteins are able to discriminate atoms by their difference in the number of neutrons. As shown in the molecular dynamic simulations presented in the results section (Figure 4, Video S1); Li^+^ ions have to first exchange the water molecules of their hydration shell with protein atoms and then translocate between several local potential grooves within the protein structure before being rehydrated on the other side of the membrane. Both for dehydration/rehydration and translocation, ions must cross successive local energy barriers. The lighter ^6^Li isotope—which has a greater vibrational velocity and therefore, higher energy than ^7^Li - has a smaller barrier to cross for each step (Figure 5). In this context, the global acceleration that we measure for ^6^Li is the sum of all the elementary differences for each microscopic transport steps between two energy minima for Li within the protein. Summing up these effects all along the translocation pathway will result in the measured rate differences that account for the isotopic effects observed in this study.

An interesting question concerns the possible entropic contribution of the protein conformational changes to this isotopic effect. This effect is difficult to quantify but while existing theoretically, it should not be a significant part of the measured isotopic transport effect. The reason is that NHEs are large proteins with a complex cycle of transport, in which the entropy associated with the protein (*plus* lipids and water) conformational changes should be orders of magnitude above that of isotopic lithium transport. Indeed, the calculated molecular mass of a NHE1 protomer is 90.763 kD and the MD simulation ran in this study with a dimeric NHE1 (lipids; protein and water/ions) contains 176 901atoms. If we assume that only ∼10% of the transporter’s structure would undergo significant conformational changes during the transport cycle that would correspond to an entropic term several orders of magnitude above the mass difference between ^6^Li and ^7^Li. Consequently, we expect the entropy associated with the conformational changes of NHE1 transport to be not significantly different between the two Li isotopes and consider that the difference in transport kinetics is instead caused by the sum of microscopic effects (hydration/dehydration/rehydration; vibrational energies of the isotopes within the protein …), as described before. Indeed, we observe these effects in the timescale of our simulation (250ns) that is too short to see large conformational changes. Of note; however, such conformational changes may become significant for transport when considering very small membrane channels, such as those produced by bacterial toxins or ionophores. This will be worth investigating in further studies.

Another important implication for transport is that the Li isotopic analyses of dose responses reveal the positive cooperativity for ^6^Li^+^. As no cooperative kinetics could be observed for external cations in NHE1 transport, isotopic measurements of transport can be established as a novel experimental method to probe the molecular mechanisms of ion channels and transporters with an unprecedented resolution.

The kinetic results presented here, show that Li isotopic fractionation persists as long as ion transport is operating. This has interesting potentialities for organ physiology. Indeed, in kidneys, apical NHEs (NHE2, NHE3) are constantly active to reabsorb Na^+^ and extrude H^+^ in order to catalyze bicarbonate reabsorption. Thus, our data may very well explain the range found experimentally in sheep kidney (Figure 1A). As neurons, astrocytes and the blood brain barrier undergo an intensive ion transport activity; our results also provide clues for the enhanced behavioral effects of ^6^Li that is reported in the literature in model animals. In conclusion, measuring Li isotopes and their transport in soft tissues could be a new field of interest to explore kidney, brain—and possibly other organs function or pathological evolution.^37^ In addition, it could be in principle possible to build organ inspired industrial systems using solid-supported cell cultures coupled to adequate tubing and valves to separate Li isotopes.

Implications in geoscience can be developed as well. Indeed, ^6^Li^+^ enrichments (low δ^7^Li values) have been observed in marine biogenic carbonates, especially over the last 70 Ma period and during short-term climatic or mass extinction events.^14^^,^^38^^,^^39^^,^^40^^,^^41^^,^^42^ *In vivo*, CaCO_3_ mineral precipitation in cells produces large amounts of H^+^ ions that are eliminated in part by NHEs, which in turn enrich the precipitate in ^6^Li^+^. Ion transport activities are influenced by the external conditions (pH, temperature, and Li concentrations). Consequently, our results provide an alternative interpretation of fossil data, more closely related to biological activity variations. Hence the relationship between climate and continental chemical weathering (which removes CO_2_ from the atmosphere and delivers aqueous Li to the ocean) was likely much less strengthened than initially proposed (e.g.^43^). Finally, as the genes encoding ubiquitous Na^+^/H^+^ exchangers are thought to have encoded one of the primary ion transport structures of the first protocellular systems,^44^ our study also suggests that biological Li isotopic fractionation could be a hallmark of life.

### Limitations of the study

While we clearly found that the background low isotopic fractionation follows a mechanism mediated by ion channels, we did not molecularly identify these channels one by one in the used cell line. We considered that at this step it was too much out of the scope of the present work that mainly focused at exploring how ubiquitous NHE exchangers were responsible for lithium biological fractionation.

## STAR★Methods

### Key resources table

**Table:** 

REAGENT or RESOURCE	SOURCE	IDENTIFIER
**Chemicals, peptides, and recombinant proteins**		

G418	Sigma-Aldrich	Cat#A1720
Hygromycin B	Sigma-Aldrich	Cat#H3274
Cariporide	Sigma-Aldrich	Cat#SML1360
BCECF/AM	ThermoFisher Scientific	Cat#B1170
HCl 32% Ultrapur	VWR	Cat#83878.290
HNO3 Ultrapur	VWR	Cat#83879.290
H2O2	ThermoFisher Scientific	Cat#11692809

**Critical commercial assays**		

Lipofectamine 3000	ThermoFisher Scientific	Cat#L3000-008
NucleoBond Xtra Midi EF	Macherey Nagel	Cat#740420.50

**Deposited data**		

Structure of a human NHE1-CHP1 complex under pH 7.5, bound by cariporide	https://doi.org/10.2210/pdb7DSX/pdb	7DSX

**Experimental models: Cell lines**		
NHE-deficient fibroblasts	Pouysségur et al.^19^	PS120
NHE-1 expressing fibroblasts	Counillon et al.^21^	NHE1
NHE-2 expressing fibroblasts	Counillon et al.^21^	NHE2
NHE-3 expressing fibroblasts	Counillon et al.^21^	NHE3
NHE-6 expressing fibroblasts	In this study	NHE6
NHE-7 expressing fibroblasts	Milosavljevic et al.^22^	NHE7

**Recombinant DNA**		

pECE vector	addgene	26453
pIRES neo vector	Clontech/Takara	Gen Bank U89673
pIRES hyg3 vector	Clontech/Takara	631620

**Software and algorithms**		

UPSYLON C++ library	Bouret	https://doi.org/10.5281/zenodo.7575554
CLIFF C++ home-made fitting software	Bouret	https://doi.org/10.5281/zenodo.7576014
Gromacs MD software	https://www.gromacs.org/Abraham, M. J. et al. SoftwareX 1–2, 19–25 (2015).	
Charmm36 Force Field	Huang, J. & MacKerell, A. D.. J. Comput. Chem. 34, 2135–2145 (2013).	
Charmm-Gui membrane builder	https://www.charmm-gui.org/?doc=input/membrane.bilayer J. Comput. Chem. 29:1859-1865	

**Other**		

Atomic absorption spectrometer Pin AAcl 900Z	Perkin elmer	
Atomic absorption spectrometry Li Lamp lumina	Perkin elmer	N305-0142
Orbit mini	Nanion	
MC-ICP-MS Neptune *Plus*	ThermoFisher Scientific	

### Resource availability

#### Lead contact

Further information and requests for resources and reagents should be directed to and will be fulfilled by the lead contact, Laurent Counillon (Laurent.Counillon@univ-cotedazur.fr).

#### Materials availability

All data are available in the manuscript text and supplemental information. This study did not generate new unique reagents, all the reagents and molecules used in this work are commercially available. Plasmids generated in this study are not deposited, all cell lines and/or plasmids are available on request.

### Experimental model and subject details

#### Cell lines

The PS120 cell line is the starting line for all the other lines used in this study. This cell line was obtained in 1984^19^ by genetic selection where Na+/H+ transporter was used as a H+ killing tool. Lung fibroblasts from female chinese hamster (*Cricetulus griseus*) were loaded with LiCl and subsequently incubated in a choline chloride medium devoided of Na+ or Li+ at pH 5.5. This procedure result in a rapid cytoplasmic acidification, killing the majority of the cell population. 0.1% of cells survived and were all deficient in Na+/H+ activity. Cells are maintened at 37°C in an incubator delivering a humid atmosphere with 5% C02 in classical DMEM, with 7.5% heat inactivated Fetal calf serum and 100U/ml penicillin/streptomycin.

Stably transfected cell lines were cultured in the same medium with the addition of the corresponding selection antibiotic, G418 (750μg/ml) or Hygromycin B (500μg/ml).

### Methods details

#### Sheep experiment

The complete design of the feeding experiment for sheep is described in refs.^1^^,^^45^ Briefly, three Suffolk cross lambs (2 males and 1 female), were raised at the Teagasc Grange Beef Research Centre, Dunsany, Co. Meath, Ireland. The experimental diet consisted of 76% (wet weight basis) pelleted maize concentrate and 24% (wet weight basis) maize silage. The animals were kept on the experimental diet for 231 days. The heterogeneity of this diet has been evaluated by measuring three different aliquots. Upon completion of the experiment (April 3rd, 2007), they were transported to the Teagasc, Ashtown Food Research Center, where their organs were excised and immediately freeze dried. All procedures employed in this study were in accordance with EU regulations concerning animal welfare and use. The experiment was carried out with the approval of Teagasc, the Irish Agriculture and Food Development Authority. Between 100 and 200 mg of tissue samples were dissolved by microwave digestion using concentrated HNO_3_. After evaporation to dryness, samples were dissolved alternatively in concentrated HCl and in aqua regia at 150 °C for 2 weeks, to obtain complete dissolution. All samples were then dried and dissolved in 1.0 M HCl for solid–liquid chromatography as described in the section “lithium purification for isotopic analyses”.

#### Cell culture

Fibroblasts from the PS120 cell line^19^ were used either as control cells or stably transfected with the pECE expression vector containing NHE1, NHE2 or NHE3 cDNAs^21^ to measure the isotopic fractionation mediated by these exchangers. As pECE vector does not have antibiotic resistance, it is by repeated procedures of functional tests (cytoplasmique acid recovery) that the cells were selected. At day 2, transfected cells were submitted to an ammonium loading in a CO2 free incubator.^46^ Cells were then rapidly rinsed with a choline solution leading to a rapid acidification of their cytoplasm around pH 5. A solution containing 140mM NaCl was then added, only cells expressing a functionnal exchanger at the plasma membrane survived and grown. After 3 weeks of this selection procedure (every 3 days) all the cells are expressing the transfected NHE. PS120 cells were also transfected using lipofectamine 3000 with NHE6 (pIRES neo vector, clontech) or NHE7^22^ (pIRES Hyg3 vector, clontech) and selected with their corresponding antibiotics G418/Hygromycine B. As these 2 NHEs are expressed in intracellular membranes, plasma membrane NHE activity was not present in selected stably expressing cells. To get plasma membrane expression of these NHEs, once antibiotics selection has been completed, cells have been submitted to sublethal UVs exposition to generate random mutations. Mutated cells were then exposed to repeated acute acidifications with extracellular NaCl. This functional selection allows us to generate cell lines expressing wild type NHE6 or NHE7 at the plasma membrane (resequencing cDNA shows no mutation).

#### Methods used for quantifying Li isotopes transport

All experiments of lithium uptake were conducted on cells acidified using the ammonium prepulse technique.^46^ Instead of having extracellular sodium to recover from this cytoplasmic acidification, cells were incubated in a isotonic lithium solution, so that the NHEs could only use Li+ as coupling cation to extrude intracellular H+ ions.^47^ These experiments were performed without CO_2_ and bicarbonate to ensure that the only active pH regulating transporters are NHEs. Cells seeded on 24-well plates were acidified using the NH4+ loading technique.^46^ Measurements were performed at 37°C by incubating acidified cells in an uptake medium with various concentrations of LiCl (Sigma Aldrich) and the impermeant choline-chloride cation to maintain osmolarity. Results shown in Data S3 we obtained using cariporide (Sigma-Aldrich) a specific NHE inhibitor. Cariporide (10μM) was added in the LiCl uptake medium.^47^ After the indicated durations, uptake was stopped by four rapid rinses in ice-cold PBS in order to eliminate extracellular Li. Cells were solubilized in 25% nitric acid (trace metal grade 70%, Fisher Scientific) and aliquoted for Li concentrations and for Li isotope analyses. Total intracellular Li^+^ concentrations were measured using Atomic Absorption Spectrometry (AAS, Perkin Elmer Pin AAcl 900Z, in LP2M) and by MC-ICP-MS during isotopic analyses (Thermofischer Sci. In ENS-Lyon, National CNRS-INSU Isotopic Platform), with a volume of 2.6 103 cubic μm/cell. Total protein content of each well was measured using Biorad DC protein assay.

#### NHE1 activity measurement by fluorescence pH imaging for pH recovery

Cells were loaded with a ratiometric pH-sensitive fluorescent dye BCECF/AM which is pH sensitive when excited at 490 and possesses a 445 nm isobestic point.

We recorded images with an imaging set consisting of an inverted microscope coupled to a high sensitivity video camera. The system is equiped with 450 nm and 490 nm narrow band interference filters paired with appropriate quartz neutral-density filters for excitation. To measure NHE1 activity, cells were incubated for 1h in ammonium solution without bicarbonate nor CO2; BCECF was added to a final concentration of 5μM for 5 min and then rinsed with the same ammonium solution. Cells were placed on the microscope and baseline was established with several images. Cells were then exposed to the choline chloride solution with a rapid perfusion system, resulting in a rapid drop of fluorescence corresponding to the acidification. Once the pH stabilized we applied a solution containing counterion. like Na+ or Li+ to measure the pH recovery rate of the exchangers. At the end of each experiment a calibration was performed using 140 mM K+, 5 mM nigericin solutions ranging from pH 6.5 to 7.4. Fluorescence measurements were collected and intracellular pH values for each cell or region of interest was calculated following this equation: pH = pKa + (Log (R-Rmin)/(R_max_-R))x F_min(λ2)_/F_max(λ2)._

As we have included calibration after each pH measurement we directly calculate the difference between the value measured and the calibration. To have more detailed procedure for data treatment see.^47^

#### Lithium purification for isotopic analyses

After the experiments, collected cells and lysates were treated with concentrated and trace cleaned acids, and Li extracted and purified in a clean laboratory for preparing them to isotope analyses. In brief, cells were dissolved using concentrated HNO_3_, and H_2_O_2_, followed by reverse aqua regia, in order to obtain a complete dissolution. All samples were then dried and dissolved in titrated ultrapure 1.0 M HCl, and centrifuged. The dissolution was considered as complete if no residue was visible at this step. Then, Li separation and purification were performed using 8 cm high AG50-X8 cation exchange resin columns, following a well-established procedure.^5^^,^^7^ Each sample was passed through the cationic exchange resin columns twice, in order to fully purify the Li fractions from the sample matrix. The solutions were dried and hot residues dissolved in 0.05M HNO3 for isotope analyses.

#### Lithium isotope analyses by MC-ICP-MS

For all samples, cells and solutions, Li isotope analyses were performed using the Neptune *Plus* MC-ICP-MS (Multi Collector Ion Coupling Plasma Mass Spectrometry) available in the National CNRS-INSU Facilities platform at ENS-Lyon. The corresponding configuration use an Aridus II desolvating system, and specific high sensitivity cones. Thus, the sensitivity is about 1V/ppbLi, with a small, constant, and regularly monitored memory effect (typically less than 1.5% of the ^7^Li signal), necessary to measure low Li level biological materials. The standard bracketing technique is used along with a systematic blank correction before each sample and standard, in order to correct from the instrumental isotope fractionation. Several reference materials are run during each measurement session, typically Li7-N, Seawater and LiCl Sigma-Aldrich solution.^7^ When possible, analytical replicates were performed in order to verify the representative nature of the measured isotope ratio. Finally, full replicates were measured including the whole protocol from the Li uptake by cells, dissolution, and Li purification and Li isotope analyses, in order to quantify the reproducibility. All corresponding uncertainties are reported in the Supplementary Tables.

#### Electrophysiological measurements of lithium transport

Membrane fractions from PS120 cells were prepared using mechanical disruption by the nitrogen decompression method (Parr cell disruption vessel). This step was followed by sucrose fractionation, solubilization in an appropriate non-ionic and non-denaturing detergent (Active Motif, USA), and incorporation into artificial lipid bilayers (Orbit mini, Nanion, Germany). Briefly, lipid bilayers are formed using 1,2-diphytanoyl-sn-glycero-3-phopsphocholine (DPHPC) lipid (10 mg/mL in octane), and membrane fractions were reincorporated by applying the appropriate amount of proteins to the upper chamber to elicit single channel activity. Single channel recordings were performed in a symmetrical solution (140 mM Li-acetate, pH7.4) to elicit only Li+ currents.

#### Molecular dynamics (MD) simulations

The system composed of the Structure of the dimer human NHE1-CHP1 (Calcineurin B-homologous protein 1) (PDB 7DSX) embedded in a 370 POPC lipids bilayer was built with the CHARMM-GUI membrane builder tool.^48^ The ligands and chains C,D have been removed to focus on the transmembrane part. MD simulations were performed with GROMACS 2021.3^35^ with the CHARMM36 force field.^49^ The TIP3P water model configuration was used. Na and Cl ions were added to neutralize the simulation box, at a minimum concentration of 120 mM. 3 Na+ (MD1) or 5 Na+ (MD2) ions have been replaced by Lithium ions close to the channel. The total number of atoms (protein + solvent + ions) was 176 901. The systems were equilibrated with a standard six-step process provided by CHARMM-GUI. A cutoff distance of 1.2 nm was used for generating the neighbor list and this list was updated at every step. Long-range electrostatic interactions were calculated using the particle mesh Ewald summation methods (PME).^50^ Periodic boundary conditions were used. During equilibration, the protein molecule was restrained. All bonds were constrained by the LINear Constraints Solver (LINCS) constraint algorithm. During the production run, the Nose-Hoover thermostat and Parrinello–Rahman barostat stabilized the temperature at 310 K and pressure at 1 bar, respectively. Each simulation was performed for 250ns, and coordinates were saved every 100 ps. Structure analysis were done using GROMACS utilities.

### Quantification and statistical analysis

The detailed table data are present in supplemental information. All the data corresponding to figures are presented, the exact value of each “n” is mentioned in each table. Error bars represent +/- SEM.

#### Fitting method

From the derived model presented in the supplemental information, two sets of data were fitted, namely the lithium intake [Li](t) and the lithium fractionation delta(t), with the same set of parameters driving the proposed differential system. For this, the least-squares fitting method through the classical Levenberg-Marquardt algorithm was used.

As each data point is to be compared with the time integration of a significant number of differential equations (themselves depending on the fitting parameters), we designed a specific C++ code to perform an incremental computation of the fitting guesses in a reasonable amount of CPU time, and then to feed the least-squares process.

To integrate each required time step for a given set of parameters, an adaptive time-step differential equation solver was implemented, using the 4th order Dormand-Price algorithm (as used by Matlab(tm) 'ode45' function).

Finally, an iterative search of the fitting parameters was performed, while discriminating the effect of each variable on [Li](t) only, delta(t) only, or on both functions. The code is available online (key resources table, software and algorithms). See the supplemental information for a full explanation of the methodology used for mathematical modeling the kinetics and dose-response of Lithium isotopic fractionation.

## Data Availability

• Data tables are presented in supplemental information. Structure data used for the simulation is NHE1 are deposited, DOi is in the key resources table. • All original code has been deposited at Zenodo and is publicly available as of the date of publication. DOIs are listed in the key resources table. • Any additional information required to reanalyze the data reported in this paper is available from the lead contact upon request.
