# Supplementary material for: Biological fractionation of lithium isotopes by cellular Na+/H+ exchangers unravels fundamental transport mechanisms
Source: iScience. 2023 May 15;26(6):106887. doi: 10.1016/j.isci.2023.106887 (PMC10265516; doi:10.1016/j.isci.2023.106887)
Supplement: Document S1. Figures S1–S3 and Data S1–S7 [file mmc1.pdf]

iScience, Volume *26*

Supplemental information

Biological fractionation of lithium isotopes by cellular Na^+/H^+ exchangers unravels fundamental transport mechanisms

Mallorie Poet, Nathalie Vigier, Yann Bouret, Gisèle Jarretou, Romain Gautier, Saïd Bendahhou, Vincent Balter, Maryline Montanes, Fanny Thibon, and Laurent Counillon

SUPPLEMENTAL TEXT AND FIGURES

1. Tables of results for Li transport

Data S1. Kinetic Li and Li isotopic active transport through membranes of NHE1 equipped cells. For all experiments, the external solution Li concentration was 15mM and its δ^7Li value is 15.0‰ +/-0.3‰ ($2\sigma_n$, n=10). n is the number of full replicates (from experiment to isotope analysis). Associated to figure 2d. Related to Star "Methods used for quantifying Li isotopes transport"

Cell line	T (°C)	Ext Li (mM)	Uptake duration (s)	Intracell Li (mM)	$2\sigma_n$	δ^7Li (‰)	$2\sigma_n$	n
NHE-1	37	15	0	0.04				1
NHE-1	37	15	5	5.11	2.2	1.00	0.44	2
NHE-1	37	15	10	10.49	5.3	0.83	0.30	2
NHE-1	37	15	15	14.68	3.9	1.31	0.72	4
NHE-1	37	15	30	24.22	2.8	1.57	0.19	3
NHE-1	37	15	60	35.19	3.7	2.55	0.94	9
NHE-1	37	15	180	66.17	29.3			1
NHE-1	37	15	300	101.38	10.1	7.54	0.40	1
NHE-1	37	15	900	108.81	10.9	12.64	0.40	1
NHE-1	37	15	3600	88.52	21.9	13.24	1.73	3

Data S2. Kinetic Li passive transport through membrane of cells which do not express any NHE (PS120 or NHE-Null cell). For all experiments, the external solution Li concentration was 15mM. Associated to figure 2c.Related to Star "Methods used for quantifying Li isotopes transport"

Cell line	T (°C)	Ext Li (mM)	Uptake duration (s)	Intracell Li (mM)	n
PS120	37	15	15	0.75	3
PS120	37	15	30	0.77	3
PS120	37	15	60	1.64	3
PS120	37	15	180	4.03	3
PS120	37	15	360	7.10	3
PS120	37	15	600	9.61	3
PS120	37	15	1200	14.3	3
PS120	37	15	1800	15.5	3
PS120	37	15	2400	19.6	3
PS120	37	15	3600	24.7	3

Data S3. Dose Response of Li and Li isotopes active (NHE1) and passive (Cariporide inhibitor) transport through membrane of cells equipped with NHE1. For all experiments, the duration of the Li uptake was 60 s. The external solution δ^7Li value is 15.0‰ +/-0.3‰ ($2\sigma_n$, n=10). Associated with part "pH induced and dose-response of Li isotope fractionations reveal cooperativity "from result section. Related to Star "Methods used for quantifying Li isotopes transport"

Cell line	T (°C)	Ext Li (mM)	Intracell Li (mM)	$2\sigma_n$	δ^7Li (‰)	$2\sigma_n$	n
NHE1	37	0	0.04	0.004			1
NHE1	37	0.3	3.16	0.32	5.37	0.02	2
NHE1	37	1	6.39	4.9	3.86	1.37	4
CARIPORIDE*	37	1	0.10	0.004	8.89	0.48	2
NHE1	37	3	11.62	8.0	3.92	1.34	5
CARIPORIDE*	37	3	0.19	0.04	8.26	1.49	2
NHE1	37	15	35.19	3.7	2.55	0.94	9
NHE1	20+	15	35.94	11.4	2.71	0.24	1
NHE1	37	30	30.44	23.0	1.88	0.40	4
CARIPORIDE*	37	30	1.51	0.33	6.72	0.17	2
NHE1	37	60	11.85	0.59	1.43	0.25	2
NHE1	37	90	12.57	0.10	1.09	0.23	2
NHE1	37	120	1.81	0.18	1.61	0.02	1

*Cariporide inhibits the Li active transport by NHEs, as evidenced by the low intracellular Li measured at the end of the experiments.

+This experience has been performed at 20°C for comparison

Data S4. Li and Li isotopic active transport by NHE1 equipped cells when the external solution contains variable amounts of Na. For all experiments, the duration of the Li and Na uptake was 60 s. For all experiments, the external solution Li concentration is 15mM and its δ^7Li value is 15.0‰ +/-0.3‰ ($2\sigma_n$, n=10). Associated to figure 3e & f.

Related to Star "Methods used for quantifying Li isotopes transport"

Ext Na (mM)	T (°C)	Ext Li (mM)	Intracell Li (mM)	$2\sigma_n$	δ^7Li (‰)	$2\sigma_n$	n
0	37	15	35.19	3.7	2.55	0.94	9
10	37	15	32.26	1.71	2.51	0.20	3
30	37	15	28.90	2.59	2.38	0.36	3
60	37	15	23.23	5.25	3.31	0.25	3
105	37	15	15.35	1.53	2.76	0.02	1

Data S5. Li and Li isotopic active transport by NHE1 equipped cells when the internal solution contains variable amounts of protons (pH different). For all experiments, the duration of the Li uptake was 60 s. For all experiments, the external solution Li concentration is 15mM and its δ^7Li value is 15.0‰ +/-0.3‰ ($2\sigma_n$, n=10). **Associated to figures 3a & b.**

Reated to Star "Methods used for quantifying Li isotopes transport"

Intracell pH*	T (°C)	Ext Li (mM)	Intracell Li (mM)	$2\sigma_n$	δ^7Li (‰)	$2\sigma_n$	n
7.30	37	15	1.10	0.05	16.7	0.9	2
6.85	37	15	2.79	0.65	7.6	2.0	3
6.51	37	15	17.66	2.80	2.7	0.1	3
6.30	37	15	26.25	4.57	2.1	0.4	3
6.24	37	15	34.52	2.66	2.0	0.1	2
6.13	37	15	35.19	3.7	2.5	0.9	9

*intracellular pH were reached using various NH_4Cl uptake times, and was estimated using the calibration described in the Method section.

2. Figure S1: Li^+ currents in lipid bilayers :

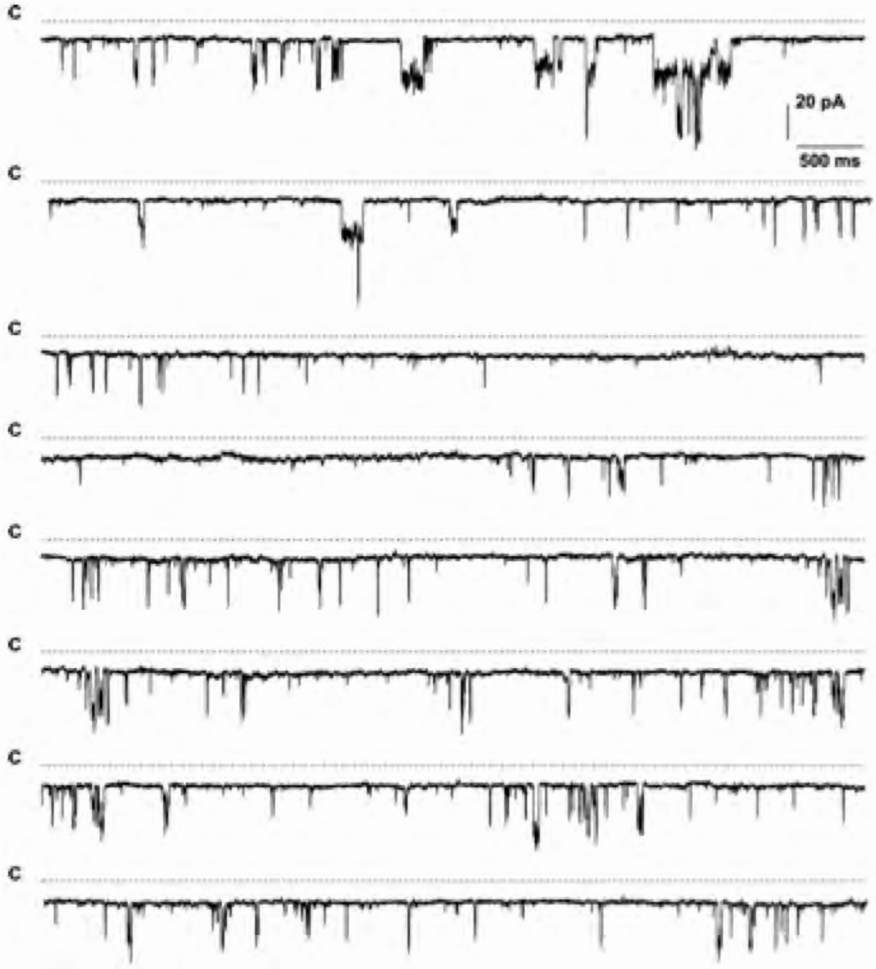

14

NHE-1-expressing PS120 membrane fragments were incorporated into lipid bilayers and current recordings were performed at various continuous potentials. We show a representative current gating from a closed state (C) elicited by application of a potential of -100 mV. Single channel opening events appear as downward deviations of the baseline, which correspond to Li currents in the range of the scale (20 pA) and of various frequencies and durations (from several ms to >100ms). Associated with part "Biological Li isotope fractionation is driven by kinetics" from result section. Related to Star Methods "Electrophysiological Measurements of Lithium Transport".

3. Supplementary Kinetic Model Construction

3.1. CELLULAR SYSTEM WITH ISOTOPIC FRACTIONATION

We consider a cell prepared according to the protocol described in the main manuscript, and we deal with a cell model as described by Figure S2A. The cytosolic concentrations and variables are named without any specific index, and the outer concentrations and variables are named with a 'out' index. Hence, the variables of interest are:

- Λ as the total lithium concentration,
- E_m as the membrane potential (see §3.3 for its derivation),
- $[\,^6Li^+]_{out} = \epsilon_6 \Lambda_{out}$, where $\epsilon_6 \simeq 0.076$ is the natural abundance of $^6Li^+$ in the outer lithium solution,
- $[\,^7Li^+]_{out} = \epsilon_7 \Lambda_{out}$, where $\epsilon_7 \simeq 0.924$ is the natural abundance of $^7Li^+$ in the outer lithium solution.

The table in Data S6 sums up the different initial values that we need to start a kinetic model.

Initial Value	Outer Medium	Cytosol
$[Li^+]$	Λ_{out}	$\Lambda = 0$
$[\,^6Li^+]$	$\epsilon_6 \Lambda_{out}$	0
$[\,^7Li^+]$	$\epsilon_7 \Lambda_{out}$	0
$[K^+]$	10 mM	140 mM
$[Cl^-]$	140 mM	20 mM
$\delta^7 Li^+$	14.57	N/A
E_m	–	-40 mV

Data S6: Summary of values accounting for the initial cell state.
Related to Star Methods "Fitting Method"

3.2. TRANSPORTING ISOTOPES BY AN ACTIVATED PROCESS

A. Description

As referred to in the main manuscript, a recent study showed how one monovalent cation is driven across one NHE monomer with the occurence of a local flip, leading the cation from a nanoscopic groove close to the outer medium (*ie* the "outer groove", outlined in blue on the left of Figure S2B) to a nanoscopic groove close to the cytosol (*ie* the "cytosolic groove", outlined in orange on the right of Figure S2B). We assume that this molecular flipping mechanism is the rate determining step of the lithium translocation, and the free energy accompanying this transformation is sketched on Figure S2B, where all the relevant free energies are reported. We classically consider that there is no significant energy well near the unbounded free energy maximum.

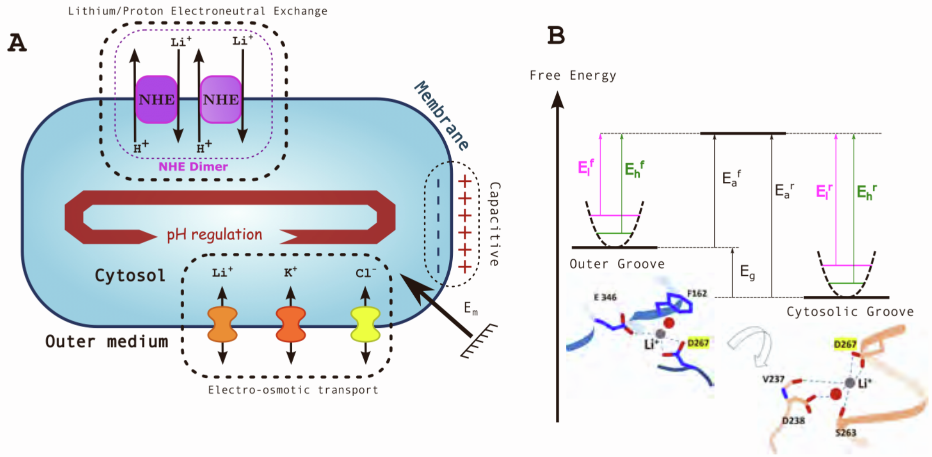

Figure S2: Scheme of isotopic transport by an activated process. **A**. *Representation of the different kinetic elements.* **B**. *Schematic translocation from the outer groove to a more stable cytosolic groove within one NHE monomer. The absolute free energy gain is E_g, while the absolute activation energies are shown as E_a. A light element ($^6Li^+$ in magenta) starts from a quasi-harmonic state (see § 3.2 B) and has a forward activation energy E_l^f and a backward activation free energy E_l^b. A heavy element ($^7Li^+$ in green) starts from a quasi-harmomic state and has a forward activation energy E_h^f and a backward activation energy E_h^b.*
Related to Figures 4 and 5

B. Harmonic Approximations and Reduced Masses

We assume here that the energy minima are approximated by two harmonic potentials with a stiffness of k_r for the "reactants" (outer groove) and k_p for the "products" (cytosolic groove). Accordingly, the fundamental energy levels E_r and E_p are respectively :

$$\begin{cases} E_r(\mu) &= \frac{1}{2}\hbar\sqrt{\frac{k_r}{\mu}} \\ E_p(\mu) &= \frac{1}{2}\hbar\sqrt{\frac{k_p}{\mu}} - E_g, \end{cases} \tag{1}$$

where μ is the reduced mass of the interacting particules and $\hbar$ is the reduced Plank's constant. Using this result and the notation of § 3.2 A and Figure S2, we shall now derive the differences in thermodynamics and kinetics, depending on the reduced masses.

Moreover, the reduced mass is always close to the mass of the lightest particle that is involved in the chemical transformation. Hence we expect μ_l to be the mass of $^6Li^+$ and μ_h to be the mass of $^7Li^+$.

C. Isotopic Thermodynamics

For a given reduced mass μ, the total difference of energy between the states can be described as:

$$\Delta E(\mu) = E_p(\mu) - E_r(\mu) = -E_g + \frac{\hbar}{2\sqrt{\mu}}\left(\sqrt{k_p} - \sqrt{k_r}\right). \tag{2}$$

If we now use μ_l for the lighter system and μ_h for the heavier system, the difference of energy differences is:

$$\underbrace{\Delta E(\mu_l) - \Delta E(\mu_h)}_{\Delta\Delta E} = \frac{\hbar}{2}\left(\sqrt{k_p} - \sqrt{k_r}\right)\underbrace{\left(\frac{1}{\sqrt{\mu_l}} - \frac{1}{\sqrt{\mu_h}}\right)}_{>0}. \tag{3}$$

- If the species senses the same environment ($k_r \simeq k_p$), then the energy difference is the same and there is no influence of the mass on the thermodynamics.
- Otherwise, a slight change in composition may occur.
  - If the outer groove potential well is less stiff than the extracellular one ($k_p < k_r$), then there will be an excess of lighter products ($\Delta\Delta E < 0$).

– Conversely, if the cytosolic groove potential well is stiffer than the extracellular one. ($k_r < k_p$), then there will be an excess of heavier products ($\Delta\Delta E > 0$).

D. Isotopic Kinetics

Using the previous notations, the forward and backward activation energies are respectively:

$$\begin{cases} E_a^f(\mu) &= E_a - E_r(\mu) \\ E_a^b(\mu) &= E_a - E_p(\mu) \end{cases} \tag{4}$$

Accordingly, we deduce the respective apparent first order rate constants:

$$\begin{cases} k_a^f(\mu) &= k_0^f e^{\left[\frac{E_r(\mu)}{k_BT} - \frac{E_a}{k_BT}\right]} \\ k_a^b(\mu) &= k_0^b e^{\left[\frac{E_p(\mu)}{k_BT} - \frac{E_a}{k_BT}\right]}. \end{cases} \tag{5}$$

Let us now evaluate the ratio of the lighter forward rate over the heavier forward rate:

$$\begin{cases} \dfrac{k_a^f(\mu_l)}{k_a^f(\mu_h)} &= \exp\left[\dfrac{E_r(\mu_l) - E_r(\mu_h)}{k_BT}\right] \\ &= \exp\left[\dfrac{\hbar\sqrt{k_r}}{2k_BT}\underbrace{\left(\dfrac{1}{\sqrt{\mu_l}} - \dfrac{1}{\sqrt{\mu_h}}\right)}_{>0}\right] \\ &= \kappa_f > 1. \end{cases} \tag{6}$$

Similarly, the ratio of the lighter backward rate over the heavier backward rate can be written:

$$\begin{cases} \dfrac{k_a^b(\mu_l)}{k_a^b(\mu_h)} &= \exp\left[\dfrac{\hbar\sqrt{k_p}}{2k_BT}\underbrace{\left(\dfrac{1}{\sqrt{\mu_l}} - \dfrac{1}{\sqrt{\mu_h}}\right)}_{>0}\right] \\ &= \kappa_b > 1. \end{cases} \tag{7}$$

E. Conclusions

When two isotopes undergo the same transformation in a potential energy landscape, some differences occur in both thermodynamics and kinetics. Actually, the lighter isotope preferentially stays in the less constraint spaces (a.k.a the heavier isotope

preferentially stays in the most constraint spaces), and always has a faster way to reach its equilibria.

Moreover, if two isotopes undergo a set of transformations leading to different potential wells with stiffness $k_{p,j\in[1,n]}$, then the apparent initial speedup κ_n^f is expressed by:

$$\begin{aligned}
\kappa_{f,n} &= \exp\left[\frac{\hbar}{2k_BT}\left(\frac{1}{\sqrt{\mu_l}}-\frac{1}{\sqrt{\mu_h}}\right)\left(\sum_{j=1}^{n}\sqrt{k_{p,j}}\right)\right] \\
&= \exp\left[\frac{n\hbar}{2k_BT}\left(\frac{1}{\sqrt{\mu_l}}-\frac{1}{\sqrt{\mu_h}}\right)\frac{\left(\sum_{j=1}^{n}\sqrt{k_{p,j}}\right)}{n}\right] \\
&= \exp\left[\frac{\hbar}{2k_BT}\left(\frac{1}{\sqrt{\mu_l}}-\frac{1}{\sqrt{\mu_h}}\right)\langle k_p\rangle\right]^n \\
&= \langle\kappa_f\rangle^n
\end{aligned} \tag{8}$$

3.3. ELECTRO-OSMOTIC TRANSPORT

A. Leaks arising from the electro-osmotic gradients

The net charge difference between the cytoplasm and the outer medium gives birth to a cell membrane polarisation E_m. Consequently, each ion leaks through membrane channels according to both its specific permeability and the Goldman-Hodgkin-Katz (GHK) flux equations. We sum up here the corresponding intakes for an anion A^- or a cation Q^+ whilst relying on a former formalism[29] , where k_A and k_Q are their respective full-cell permeabilities. We define:

- the reduced potential ζ, proportional to E_m and the Faraday's constant $\mathcal{F}$:
$$\zeta = \frac{\mathcal{F}E_m}{RT},$$
- the GHK weight function $\Psi(\zeta)$:
$$\Psi(\zeta) = \frac{\zeta}{1-e^{-\zeta}} \simeq 1 + \frac{1}{2}\zeta + \frac{1}{12}\zeta^2 + \dots, \tag{9}$$
- the full cell cationic leak for Q^+: :
$$\partial_t[Q^+]|_{leak} = k_Q\Psi(\zeta)\left([Q^+]_{out}e^{-\zeta} - [Q^+]\right),$$
- and the full cell anionic leak for A^-:
$$\partial_t[A^-]|_{leak} = k_A\Psi(\zeta)\left([A^-]_{out} - [A^-]e^{-\zeta}\right).$$

The net balance of all the species X_i with a charge z_i leads to the electric equation, depending on the full-cell membrane capacitance C_m:

$$C_m\partial_t E_m = \mathcal{F}\sum_i z_i\partial_t[X_i]|_{leak}, \tag{10}$$

and we define the electric sensitivity γ as:

$$\gamma = \frac{\mathcal{F}}{C_m}. \tag{11}$$

B. Application to NHE-null (PS120) cells

1. *Corresponding differential system*

As formerly shown[29] and as there is no present Na^+ in these sets of experiments, the main ions that account for the potential evolution are K^+ and Cl^-, with $k_{Cl} \approx 0.1k_K$. We obviously take into account the lithium contributions with k_7 for ${}^7Li^+$ and k_6 for ${}^6Li^+$. In water[51] , the diffusion coefficient $D_{\,^6Li^+}$ of ${}^6Li^+$ is greater than the one $D_{\,^7Li^+}$ for ${}^7Li^+$ by a ratio:

$$\frac{D_{\,^7Li^+}}{D_{\,^6Li^+}} = 0.99772 \pm 0.00026, \tag{12}$$

which is equivalent to a diffusion speed-up of ${}^6Li^+$ compared to ${}^7Li^+$ of:

$$\sigma = \frac{D_{\,^6Li^+}}{D_{\,^7Li^+}} = 1.00229 \pm 2\cdot 10^{-4} \tag{13}$$

Since the transport of species through ion channels is considered as a diffusive process, we define the ”electro-osmotic acceleration”:

$$\sigma' = \frac{k_6}{k_7}. \tag{14}$$

Finally, the NHE-null differential system is:

$$\begin{cases} \partial_t[\,^6Li^+] &= k_6\Psi(\zeta)\left([\,^6Li^+]_{out}e^{-\zeta} - [\,^6Li^+]\right) = \partial_t[\,^6Li^+]|_{leak} \\ \partial_t[\,^7Li^+] &= k_7\Psi(\zeta)\left([\,^7Li^+]_{out}e^{-\zeta} - [\,^7Li^+]\right) = \partial_t[\,^7Li^+]|_{leak} \\ \partial_t[K^+] &= k_K\Psi(\zeta)\left([K^+]_{out}e^{-\zeta} - [K^+]\right) \\ \partial_t[Cl^-] &= k_{Cl}\Psi(\zeta)\left([Cl^-]_{out} - [Cl^-]e^{-\zeta}\right) \\ \partial_t E_m &= \gamma\left(\partial_t[\,^6Li^+]|_{leak} + \partial_t[\,^7Li^+]|_{leak} + \partial_t[K^+] - \partial_t[Cl^-]\right) \end{cases} \tag{15}$$

2. *Estimation of the constants*

Associated to figures 2c, 2d and 3a.

Related to Star Methods ”Fitting Methods”.

We designed a C^{++} software to solve the differential problem (using a Dormand-Price algorithm), coupled with a Levenberg–Marquardt method to adjust the different parameters for constraints enumerated in Data S7.

Parameter	Value	Origin
Λ_{out}	15 mM	imposed concentration
$\delta^7 Li^+|_{t=60s}$	10.8	average measured value
k_{Cl}	$0.1 \times k_K$	see electrophysiology in[29]

Data S7: Experimental constraints imposed during data adjustment.
Related to Star Methods "Fitting Method"

We obtain the following estimations:

$$\begin{cases} \gamma &= (5.21 \pm 0.38) & \text{V.M}^{-1} \\ k_K &= (0.058 \pm 0.001) & \cdot 10^{-3}\ \text{s}^{-1} \\ k_7 &= (0.992 \pm 0.04) & \cdot 10^{-3}\ \text{s}^{-1} \end{cases} \tag{16}$$

and we deduced from the computation that the ratio of the lithium permeabilities is:

$$\sigma' = \frac{k_6}{k_7} = 1.003785 \pm 3 \cdot 10^{-6}, \quad \frac{1}{\sigma'} = 0.996229 \pm 3 \cdot 10^{-6} \tag{17}$$

that consistently yields a computed value of $\delta^7 Li^+ = 10.8 \pm 0.09$ at one minute of uptake, in accordance with the required constraints. The resulting adjusted curve is shown in the main manuscript on Figure 2c.

3. *Conclusion from NHE-null measurements*

The kinetic data are fully coherent with an electro-osmotic regulation of a NHE-null cell undergoing our experimental protocol. The value of the greater selectivity for $^6Li^+$ through the ionic channels agrees with a diffusive process, as hypothesised by Goldman-Hodgkin-Katz, and this is the first time to our knowledge that a measure of this physiological constant is provided.

3.4. COOPERATIVE LITHIUM TRANSPORT BY NHE

A. Model for a dimer

1. *Combinatorics of membrane species*

Let's name the bare (*ie* without any linked ion) monomer $\mathcal{E}$. Then the bare dimer $2\mathcal{E}$ offers a side a and a side b, with two binding sites on each side. We assume that each of these sites independently accepts an ion $X_1, \ldots, X_N$, to form a pre-equilibrated species as follow:

$$\forall (i,j,k,l) \in [1:N]^4, \quad X_{(i,a)} + X_{(k,a)} + 2\mathcal{E} + X_{(j,b)} + X_{(l,n)} \rightleftharpoons \left\{ \begin{array}{ccc} X_{(i,a)} & \mathcal{E} & X_{(j,b)} \\ X_{(k,a)} & \mathcal{E} & X_{(l,n)} \end{array} \right\}, \tag{18}$$

that is described as the dimeric association of two "$X \cdot \mathcal{E} \cdot X$" protomers.
For each set of N concentrations on side a, N concentrations on side b, and the total $[\mathcal{E}]$ we derive the N^4 pre-equilibria observable constants:

$$\forall (i,j,k,l) \in [1:N]^4, \quad K_{ijkl} = \frac{\left[\left\{ \begin{array}{ccc} X_{(i,a)} & \mathcal{E} & X_{(j,b)} \\ X_{(k,a)} & \mathcal{E} & X_{(l,n)} \end{array} \right\} \right]}{[\mathcal{E}]^2 [X_{(i,a)}][X_{(k,a)}][X_{(j,b)}][X_{(l,n)}]}. \tag{19}$$

Here, for the two lithium isotopes and $[H^+]$, we deal with $N = 3$ and 81 species. From now on, we note $\vec{X}_a$ the vector of concentrations on side a, and $\vec{X}_b$ the vector of concentrations on side b. Namely, we obtain:

$$\vec{X}_{a/b} = \begin{bmatrix} [H^+]_{a/b} \\ [\,^6Li^+]_{a/b} \\ [\,^7Li^+]_{a/b} \end{bmatrix} \tag{20}$$

2. *Free NHE computation*

Let $\mathcal{E}_0$ be the total concentration of NHE. The enzyme conservation writes:

$$\mathcal{E}_0 = [\mathcal{E}] + 2[\mathcal{E}]^2 \underbrace{\left(\sum_{i,j,k,l} K_{ijkl}[X_{(i,a)}][X_{(k,a)}][X_{(j,b)}][X_{(l,b)}] \right)}_{\Xi(\vec{X}_a, \vec{X}_b)}, \tag{21}$$

leading to:

$$[\mathcal{E}] = \mathcal{E}_0 \frac{2}{1 + \sqrt{1 + 8\mathcal{E}_0 \Xi(\vec{X}_a, \vec{X}_b)}} = \frac{\mathcal{E}_0}{W(\vec{X}_a, \vec{X}_b)} \tag{22}$$

3. *Conditional exchange (flip) rates*

Each formed dimer formally flips its content with 2×2 rates, as expected from Eq.(18). We use a two sets of rates U_{ijkl} for the "upper" protomer and V_{ijkl} for the "lower" protomer to obtain:

$$\left\{ \begin{array}{l} \left\{ \begin{array}{ccc} X_{(i,a)} & \mathcal{E} & X_{(j,b)} \\ X_{(k,a)} & \mathcal{E} & X_{(l,n)} \end{array} \right\} \underset{U_{jikl}}{\overset{U_{ijkl}}{\rightleftharpoons}} \left\{ \begin{array}{ccc} X_{(j,a)} & \mathcal{E} & X_{(i,b)} \\ X_{(k,a)} & \mathcal{E} & X_{(l,n)} \end{array} \right\} \\ \\ \left\{ \begin{array}{ccc} X_{(i,a)} & \mathcal{E} & X_{(j,b)} \\ X_{(k,a)} & \mathcal{E} & X_{(l,n)} \end{array} \right\} \underset{V_{ijlk}}{\overset{V_{ijkl}}{\rightleftharpoons}} \left\{ \begin{array}{ccc} X_{(j,a)} & \mathcal{E} & X_{(i,b)} \\ X_{(l,a)} & \mathcal{E} & X_{(k,n)} \end{array} \right\} \end{array} \right. \tag{23}$$

By combining the 81 pre-equilibria with the expression of $[\mathcal{E}]$, we deduce the formal creation rate of each species as the following example:

$$\begin{aligned} \frac{1}{[\mathcal{E}]^2} \partial_t [X_{(\mathbf{i},a)}] = \; & -[X_{(\mathbf{i},a)}] \times \left(\textstyle\sum_{jkl} \left(U_{\mathbf{i}jkl} K_{\mathbf{i}jkl} + V_{kl\mathbf{i}j} K_{kl\mathbf{i}j} \right) [X_{(j,b)}][X_{(k,a)}][X_{(l,b)}] \right) \\ & + [X_{(\mathbf{i},b)}] \times \left(\textstyle\sum_{jkl} \left(U_{j\mathbf{i}kl} K_{j\mathbf{i}kl} + V_{klj\mathbf{i}} K_{klj\mathbf{i}} \right) [X_{(j,b)}][X_{(k,a)}][X_{(l,b)}] \right). \end{aligned} \tag{24}$$

We employ this formula to write the differential evolution of each chosen species.

4. *Initial isotopic separation and dose-response*

We write the initial flip rates for the lithium isotopes:

- using no index for intracellular species and a '*out*' index for extracellular species,
- using Eq.(24) to express individual rates,

- and using a '0' index for the initial time,

and we end up with the weights:

$$w_{pq} = U_{HpHq}K_{HpHq} + V_{HqHp}K_{HqHp}, \quad p \in [6:7],\ q \in [6:7],$$

and the initial fractionation:

$$\left(\frac{[\,^7Li^+]}{[\,^6Li^+]}\right)_0 = \left(\frac{[\,^7Li^+]_{out}}{[\,^6Li^+]_{out}}\right) \times \frac{k_7\Psi(\zeta_0)e^{-\zeta_0} + [H^+]_0^2[\mathcal{E}]_0^2\,(w_{76}\epsilon_6 + w_{77}\epsilon_7)\,\Lambda_{out}}{k_6\Psi(\zeta_0)e^{-\zeta_0} + [H^+]_0^2[\mathcal{E}]_0^2\,(w_{66}\epsilon_6 + w_{67}\epsilon_7)\,\Lambda_{out}}. \tag{25}$$

We clarify this expression by defining:

$$\begin{cases} \kappa' = \dfrac{w_{76}\epsilon_6 + w_{77}\epsilon_7}{w_{66}\epsilon_6 + w_{67}\epsilon_7} \\ \\ \tan^2\omega = \dfrac{(w_{76}\epsilon_6 + w_{77}\epsilon_7)\,[H^+]_0^2[\mathcal{E}]_0^2\Lambda_{out}}{\Psi(\zeta_0)e^{-\zeta_0}}, \end{cases} \tag{26}$$

and we consequently obtain:

$$\left(\frac{[\,^7Li^+]}{[\,^6Li^+]}\right)_0 = \left(\frac{[\,^7Li^+]_{out}}{[\,^6Li^+]_{out}}\right)\frac{1}{\sigma'\cos^2\omega + \kappa'\sin^2\omega} = \left(\frac{[\,^7Li^+]_{out}}{[\,^6Li^+]_{out}}\right)\frac{1+\tan^2\omega}{\sigma' + \kappa'\tan^2\omega}. \tag{27}$$

We remind the following expression:

$$\delta\,^7Li_0^+ = 10^3\left[(1 + 10^{-3}\delta\,^7Li_{out}^+)\frac{1+\tan^2\omega}{\sigma' + \kappa'\tan^2\omega} - 1\right], \tag{28}$$

and we want to find out how the initial fractionation depends on the total extracellular lithium concentration Λ_{out}, which is equivalent to study the variations of:

$$\Xi = \frac{1+\tan^2\omega}{\sigma' + \kappa'\tan^2\omega}. \tag{29}$$

Since $\tan\omega^2 > 0$ and the experimental initial concentration ratio is smaller than the external one, we deduce that $\kappa' > \sigma'$. Accordingly, we observe that Ξ is a *decreasing* function with respect to $\tan^2\omega$, which is proportional to $[\mathcal{E}]_0^2\Lambda_{out}$:

- this expression starts from zero, meaning that the first-order in Λ_{out}, GHK pathway always imposes of fractionation related to σ',

- and this expression returns to zero for high Λ_{out}, since there is a saturation in the NHE kinetics, while the GHK pathways is always proportional to Λ_{out},

- thus, since $[\mathcal{E}]_0^2\Lambda_{out}$ is positive and starts from zero and returns to zero, it is *increasing* on an interval starting from zero.

Consequently, we directly deduce that, due to the very dimeric structure of NHE:

- there exists *an optimal total outer lithium concentration that we name* Λ_{opt} *that produces a maximum initial fractionation*,
- there is an *increasing fractionation* with respect to an *increasing total outer lithium concentration* ranging from 0 to Λ_{opt} (synergic dose-response),
- and that the experimental data shows (on Figure 2c of the main article) that the value of Λ_{opt} for the studied cells is about 100mM, and therefore allows the observation of the dose-response as described above.

5. *Dose-response morphology*

Since Ξ has a very complex expression, we choose a simplified set of parameters:

- $\epsilon_6 \ll \epsilon_7$,
- $[H^+]_{out} \ll \Lambda_{out}$,
- $\forall i, j, k, l, \quad K_{ijkl} \simeq K_0$ [see 'Thermodynamic Reduction' for a formal justification].

By a continuity argument, the real behaviour of Ξ will have the same traits than this simplified version that we call $\tilde{\Xi}$. We obtain a transformed expression with the help of a scaling factor χ_7 and a *reduced concentration* λ:

$$\begin{cases} \chi_7 &= \dfrac{w_{77}}{\Psi(\zeta_0)e^{-\zeta_0}}\sqrt{\dfrac{2\mathcal{E}_0^3}{K_0}} \\ \lambda &= (2^{\frac{3}{2}}[H^+]_0\sqrt{\mathcal{E}_0 K_0})\Lambda_{out} \\ \tilde{\Xi} &= \dfrac{1}{\sigma'}\dfrac{\left[1+\sqrt{1+\lambda^2}\right]^2 + \chi_7\lambda}{\left[1+\sqrt{1+\lambda^2}\right]^2 + \dfrac{\kappa'}{\sigma'}\chi_7\lambda} \end{cases} \tag{30}$$

The *reduced concentration* λ is a non-trivial combination of all the components acting on the fractionation efficiency, and shows in particular a proportionality to Λ_{out} and the initial acidity $[H^+]_0$. Finally, we experimentally measured $\delta\ {}^7Li^+|_{t=60s}$, which is expected to be lower that $\delta\ {}^7Li_0^+$ due to the favoured entry of ${}^6Li^+$. That is why we propose the following expression:

$$\delta\ {}^7Li^+|_{t=60s} \propto 10^3\left[(1+10^{-3}\delta\ {}^7Li^+_{out})\tilde{\Xi} - 1\right]. \tag{31}$$

A simple fit procedure yields the following values, using $\kappa' \simeq 1.0148$ (see further Eq.(41)):

$$\left\{ \begin{array}{rcl} (2^{\frac{3}{2}}[H^+]_0\sqrt{\mathcal{E}_0 K_0}) & \simeq & (46 \pm 5 \text{ mM})^{-1} \\ \chi_7 & \simeq & 12 \pm 3 \\ \delta\ {}^7Li^+|_{t=60s} & \simeq & (0.45 \pm 0.05)\delta\ {}^7Li_0^+ \end{array} \right. \tag{32}$$

Obviously, these numbers are very rough and almost heuristic estimations, but we retrieve the predicted behaviour, as shown on Figure 2c with the adjusted curve.

6. *Conclusion of the role of NHE*

We observe the following points:

- *(consistency)* without NHE, equivalent to $\omega = 0$ in Eq.(27), we retrieve the passive description, as seen in Eq.(17),
- *(cooperativity)* but with NHE and its cooperative (dimeric) action, an increasing fractionation is observed for an experimentally extended range of total lithium concentration,
- *(novelty)* which is of fundamental importance, since the same approach with the assumption that only a monomer exchanges the ions classically leads to a simple saturation function for the initial intake rate with respect to time, and no such dose-response could be predicted.

B. pH recovery

One the one hand, the extracellular pH is fixed by the experiment. But on the other hand, since the kinetic scheme depends on the intracellular $[H^+]$, other measurements were performed to monitor the pH rise after the initial acidification, and are reported on Figure S3 for different total external lithium concentrations. Since we do not include in this model the full homeostasis, we take $[H^+]$ as an experimental dependent input, and it appears that, with a good approximation, $[H^+]$ is a function of time t, with parameters $[H^+]_0$, $[H^+]_\infty$, and a recovery time t_h such that:

$$[H^+] \simeq [H^+]_0 + \left([H^+]_\infty - [H^+]_0\right)\frac{t}{t+t_h} = [H^+]_\infty + \left([H^+]_0 - [H^+]_\infty\right)\frac{t_h}{t+t_h}. \tag{33}$$

We use this equation to fit the pH recovery data as indicated on Figure S3.It is important to remember that $[H^+]_\infty$ depends on Λ_{out}. From the measurements, we use an heuristic expression for $[H^+]$ dynamics depending on Λ_{out}:

$$\begin{cases} t_h = \dfrac{A_h}{\mathrm{erf}\left(B_h \Lambda_{out}\right)} \\ A_h = 23.95 \pm 1.42 \text{ s} \\ B_h = 0.0318 \pm 0.004 \text{ mM}^{-1} \end{cases} \tag{34}$$

and

$$\begin{cases} \mathrm{pH}_0 = 5.94 \pm 0.1 \\ \mathrm{pH}_\infty = \mathrm{pH}_0 + (7.40 - \mathrm{pH}_0) \dfrac{\Lambda_{out}}{\Lambda_h + \Lambda_{out}} \\ \Lambda_h = 15.11 \pm 1.5 \text{ mM} \end{cases} \tag{35}$$

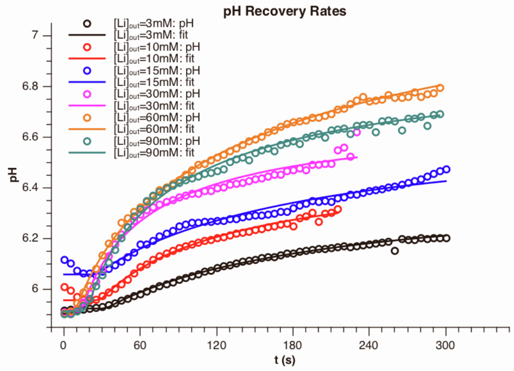

Figure S3: pH recovery kinetics at different lithium concentrations. *Adjustment [lines, according to Eq.* (33)*] of apparent experimental protons intake [open circles], for various total external lithium concentrations.* Related to Figure 3

C. Curves Adjustment

1. *Kinetic Reduction*

We are left with 81 possible species. Let's see what are their contributions based on experimental and symmetry arguments.

- We observe that **9** symmetric species are "futile": their flips produce no concentration change and they act only as inhibitors:

$$
\begin{array}{|c|c|c|}
\hline
\left\{\begin{array}{ccc} H^+ & \cdot\mathcal{E}\cdot & H^+ \\ H^+ & \cdot\mathcal{E}\cdot & H^+ \end{array}\right\} &
\left\{\begin{array}{ccc} H^+ & \cdot\mathcal{E}\cdot & H^+ \\ {}^6Li^+ & \cdot\mathcal{E}\cdot & {}^6Li^+ \end{array}\right\} &
\left\{\begin{array}{ccc} H^+ & \cdot\mathcal{E}\cdot & H^+ \\ {}^7Li^+ & \cdot\mathcal{E}\cdot & {}^7Li^+ \end{array}\right\} \\
\hline
\left\{\begin{array}{ccc} {}^6Li^+ & \cdot\mathcal{E}\cdot & {}^6Li^+ \\ H^+ & \cdot\mathcal{E}\cdot & H^+ \end{array}\right\} &
\left\{\begin{array}{ccc} {}^6Li^+ & \cdot\mathcal{E}\cdot & {}^6Li^+ \\ {}^6Li^+ & \cdot\mathcal{E}\cdot & {}^6Li^+ \end{array}\right\} &
\left\{\begin{array}{ccc} {}^6Li^+ & \cdot\mathcal{E}\cdot & {}^6Li^+ \\ {}^7Li^+ & \cdot\mathcal{E}\cdot & {}^7Li^+ \end{array}\right\} \\
\hline
\left\{\begin{array}{ccc} {}^7Li^+ & \cdot\mathcal{E}\cdot & {}^7Li^+ \\ H^+ & \cdot\mathcal{E}\cdot & H^+ \end{array}\right\} &
\left\{\begin{array}{ccc} {}^7Li^+ & \cdot\mathcal{E}\cdot & {}^7Li^+ \\ {}^6Li^+ & \cdot\mathcal{E}\cdot & {}^6Li^+ \end{array}\right\} &
\left\{\begin{array}{ccc} {}^7Li^+ & \cdot\mathcal{E}\cdot & {}^7Li^+ \\ {}^7Li^+ & \cdot\mathcal{E}\cdot & {}^7Li^+ \end{array}\right\} \\
\hline
\end{array}
\tag{36}
$$

- From well established experimental proofs[25,52] to more recent works[53] , it appears that (i) no sodium-sodium exchange occurs and that (ii) only ”matching” pairs of monomers are active. Accordingly, we assume that these behaviours stand for the lithium cases. Then we discover that **64** species are ”blocked” and act only as inhibitors.

- We have 4 ”forward” species with a flip rate f_6 (respectively f_7) when a ${}^6Li^+$ (respectively ${}^7Li^+$) is exchanged:

species	flip rates
$\left\{\begin{array}{ccc} H^+ & \mathcal{E} & {}^6Li^+ \\ H^+ & \mathcal{E} & {}^6Li^+ \end{array}\right\}$	$\begin{pmatrix} f_6 \\ f_6 \end{pmatrix}$
$\left\{\begin{array}{ccc} H^+ & \mathcal{E} & {}^6Li^+ \\ H^+ & \mathcal{E} & {}^7Li^+ \end{array}\right\}$	$\begin{pmatrix} f_6 \\ f_7 \end{pmatrix}$
$\left\{\begin{array}{ccc} H^+ & \mathcal{E} & {}^7Li^+ \\ H^+ & \mathcal{E} & {}^6Li^+ \end{array}\right\}$	$\begin{pmatrix} f_7 \\ f_6 \end{pmatrix}$
$\left\{\begin{array}{ccc} H^+ & \mathcal{E} & {}^7Li^+ \\ H^+ & \mathcal{E} & {}^7Li^+ \end{array}\right\}$	$\begin{pmatrix} f_7 \\ f_7 \end{pmatrix}$

(37)

- We have 4 ”reverse” species with a flip rate r_6 (respectively r_7) when a ${}^6Li^+$

(respectively $^7Li^+$) is exchanged:

species	flip rates
$\left\{\begin{array}{c} ^6Li^+ \ \mathcal{E} \ H^+ \\ ^6Li^+ \ \mathcal{E} \ H^+ \end{array}\right\}$	$\begin{pmatrix} r_6 \\ r_6 \end{pmatrix}$
$\left\{\begin{array}{c} ^6Li^+ \ \mathcal{E} \ H^+ \\ ^7Li^+ \ \mathcal{E} \ H^+ \end{array}\right\}$	$\begin{pmatrix} r_6 \\ r_7 \end{pmatrix}$
$\left\{\begin{array}{c} ^7Li^+ \ \mathcal{E} \ H^+ \\ ^6Li^+ \ \mathcal{E} \ H^+ \end{array}\right\}$	$\begin{pmatrix} r_7 \\ r_6 \end{pmatrix}$
$\left\{\begin{array}{c} ^7Li^+ \ \mathcal{E} \ H^+ \\ ^7Li^+ \ \mathcal{E} \ H^+ \end{array}\right\}$	$\begin{pmatrix} r_7 \\ r_7 \end{pmatrix}$

(38)

2. Thermodynamic Reduction

The determination of each K_{ijkl} would require a tremendous amount of work, far beyond the scope of the present manuscript. Indeed, one should vary each concentration, all things equal otherwise, to be able to deduce these values: this is almost impossible because of the very symmetry of the complex.
Nonetheless, we may consider that each site is not a single chemical bond, but rather an electrostatic cavity ready to accept a matching cation.
From the preceding considerations, and for the sake of simplicity and clarity, we shall set:

- for **all** i, j, k, l **of non-futile** species:

$$K_{ijkl} = K_0 \text{ in mol}^{-5} \cdot L^5 \qquad (39)$$

- and for all i, j, k, l **futile or blocked** species, $K_{ijkl} \simeq 0$. We hence report the role of unknown 73 remaining constants on apparent reverse rates, which appears to be sufficient for this model.

3. Choice and estimation of parameters

We focused on two curves, measured with 15mM of total lithium. Obviously, we kept this model as simple as possible, but still we have too many unknown parameters. Now, we look for a set of parameters:

- that allow us to reproduce the overall morphological shapes for both curves,
- and that insists on the robustness of the initial fractionation.

We proceeded as follow.

- We fix the electro-osmotic part computed from the NHE-null cells.
- We use Eq.(33) *sqq* to impose the intracellular proton concentration.
- We fix $K_0 \simeq 10 \cdot 10^{-6}\,\mathrm{mol}^{-5} \cdot L^5$ which is an average value that allows to reproduce the right shapes.
- We start from an apparent maximum rate $V_7^f = f_7 E_0 \simeq 2\,\mathrm{mM.s}^{-1}$.
- We introduce the coefficient η_f such that the apparent maximum rate for $^6Li^+$ is:

$$V_6^f = \eta_f V_7^f, \tag{40}$$

  meaning that η_f is (i) the forward isotopic speedup of NHE for $^6Li^+$, and (ii) the ”primary” constant we are looking for.

- We symmetrically use V_7^r and $V_6^r = \eta_r V_7^r$ to describe the apparent reverse maximum rate, that on the one hand exists experimentally and helps to expel the huge lithium intake, but that on the other hand is employed to mimic a part of the inhibitor species as described above.
- Since we cannot simultaneously measure the concentration and the fractionation at a given time for the same cell, and since there is a variability between experiments, we scale the computed total lithium concentration with a proportionality factor.
- We adjust the curves with the previously designed algorithm, alternating between the total (quantitative) lithium intake and the (qualitative) corresponding δ^7Li^+ up to the desired convergence.

- *Qualitative Results.* The adjusted curves are shown in the manuscript, (on Figure 2c for the total intake and for the fractionation) and show that we designed a quantitative and qualitative model. We find a very robust value for η_f:

$$\left\{ \begin{array}{lcl} \eta_f & = & \frac{V_6^f}{V_7^f} = 1.0148 \pm 0.0004 \\ \\ \frac{1}{\eta_f} & = & \frac{V_7^f}{V_6^f} = 0.9854 \pm 0.0004 \end{array} \right. \quad (41)$$

  which means, using Eq. (8), that NHE selects $^6Li^+$ about 4 times more effectively that the electro-osmotic part, and 6.5 times more effectively than the simple diffusion.

- *Quantitative Results.* But we also find out a major interest consisting in the large amount of lithium intake, since a single cell is loaded with about 100mM of total, enriched lithium in a few minutes for only 15mM of external concentration. Compared to a "passive" NHE-null cell, a simple NHE1 loaded cell loads more than 10 times the amount of $^6Li^+$ in a few minutes, which is remarkable and unprecedentedly reported.

4. Supplementary References

51. Richter, F.M., Mendybaev, R.A., Christensen, J.N., Hutcheon, I.D., Williams, R.W., Sturchio, N.C. and Beloso, Jr A.D. (2006). Kinetic isotopic fractionation during diffusion of ionic species in water, Geochimica et Cosmochimica Acta 70 277–289

52. Otsu, K., James, S., Kinsella, L., Heller, P., and Froehlich, J.P. (1993). Sodium Dependence of the Na^+-H^+ Exchanger in the
Pre-steady State J.Biol.Chem 268, 3184-3193

53. Warnau, J., Wöhlert, D., Okazaki, D., Yildiz, O., Gamiz-Hernandez, A.P., Kaila, V.R.I., Kühlbrandt, W., and Hummer, G. (2020). Ion Binding and Selectivity of the Na^+/H^+ Antiporter MjNhaP1 from Experiment and Simulation J.Phys.Chem B 124, 336-344
